# Strain variations in cone wavelength peaks *in situ* during zebrafish development

**DOI:** 10.1017/S0952523819000075

**Published:** 2019-07-30

**Authors:** Ralph F. Nelson, Annika Balraj, Tara Suresh, Meaghan Torvund, Sara S. Patterson

**Affiliations:** 1 Neural Circuitry Unit, National Institute of Neurological Disorders and Stroke, National Institutes of Health, Rockville, Maryland; 2 Department of Anatomy and Biology, George Washington University, Washington, District of Columbia; 3 Department of Biology, Washington University, St Louis, Missouri; 4 Neurobiology, University of Arizona, Tucson, Arizona; 5 Neuroscience Graduate Program, Department of Ophthalmology, University of Washington, Seattle, Washington

**Keywords:** Spectral sensitivity, Selective chromatic adaptation, Hill function, Patch electrode, Microelectrode, Xenon arc, Interference filter, Origin LabTalk, Nonlinear curve fit, Adaptation pools

## Abstract

There are four cone morphologies in zebrafish, corresponding to UV (U), blue (B), green (G), and red (R)-sensing types; yet genetically, eight cone opsins are expressed. How eight opsins are physiologically siloed in four cone types is not well understood, and in larvae, cone physiological spectral peaks are unstudied. We use a spectral model to infer cone wavelength peaks, semisaturation irradiances, and saturation amplitudes from electroretinogram (ERG) datasets composed of multi-wavelength, multi-irradiance, aspartate-isolated, cone-PIII signals, as compiled from many 5- to 12-day larvae and 8- to 18-month-old adult eyes isolated from wild-type (WT) or *roy orbison* (roy) strains. Analysis suggests (in nm) a seven-cone, U-360/B1-427/B2-440/G1-460/G3-476/R1-575/R2-556, spectral physiology in WT larvae but a six-cone, U-349/B1-414/G3-483/G4-495/R1-572/R2-556, structure in WT adults. In roy larvae, there is a five-cone structure: U-373/B2-440/G1-460/R1-575/R2-556; in roy adults, there is a four-cone structure, B1-410/G3-482/R1-571/R2-556. Existence of multiple B, G, and R types is inferred from shifts in peaks with red or blue backgrounds. Cones were either high or low semisaturation types. The more sensitive, low semisaturation types included U, B1, and G1 cones [3.0–3.6 log(quanta·μm^−2^·s^−1^)]. The less sensitive, high semisaturation types were B2, G3, G4, R1, and R2 types [4.3-4.7 log(quanta·μm^−2^·s^−1^)]. In both WT and roy, U- and B- cone saturation amplitudes were greater in larvae than in adults, while G-cone saturation levels were greater in adults. R-cone saturation amplitudes were the largest (50–60% of maximal dataset amplitudes) and constant throughout development. WT and roy larvae differed in cone signal levels, with lesser UV- and greater G-cone amplitudes occurring in roy, indicating strain variation in physiological development of cone signals. These physiological measures of cone types suggest chromatic processing in zebrafish involves at least four to seven spectral signal processing pools.

## Introduction

### The challenge of zebrafish spectral physiology

Zebrafish is a cone athlete, and ultimately, the goals in studying zebrafish color vision are to understand how the multiplicity of cone types in zebrafish serves neural circuits that process wavelength, the ecological advantages to zebrafish of such circuits, and the generation of general insights into vertebrate wavelength processing. The four cone morphologies in adult zebrafish are short singles (UV or “U” cones), long singles (blue or “B” cones), and double cones, with the principal member being a red or “R” cone and the accessory member being a green or “G” cone (Engstrom, 1960; Raymond & Barthel, 2004; Robinson et al., 1993). In adults, there is a repeating mosaic pattern of these four cones (Allison et al., 2010; Raymond & Barthel, 2004; Robinson et al., 1993). In larvae, cone morphologies are less easily identified, but opsin antibody staining reveals distinct red, green, blue, and UV cone populations, each in regular mosaic pattern (Allison et al., 2010; Suzuki et al., 2013). But screening of zebrafish cDNA libraries reveals eight cone opsin genes: *SWS1* (U); *SWS2* (B); *RH2-1*, *RH2-2*, *RH2-3*, and *RH2-4* (G1-G4); and *LWS1* and *LWS2* (R1, R2) (Chinen et al., 2003). The multiple green and red opsins are gene duplications with highly homologous sequences, so that antibodies do not readily distinguish them, yet the duplicated opsins reveal distinct spectral peaks (Chinen et al., 2003). How these 8 opsins are distributed among 4 morphological cone types and whether one or multiple opsins are physiologically active in an individual cone is not known, and further, the *in situ* physiological spectral peaks of the 8 opsins, particularly within larval cones, have yet to be measured. Detergent extracts of opsins generated in cell lines are often very close matches to patch-recorded photocurrent spectra from single cones, but sometimes not, as with red cones in zebrafish adults (Endeman et al., 2013).

Not only have eight cone opsin genes been detected in zebrafish (Chinen et al., 2003) but also the developmental courses of gene expression were followed by *in situ* hybridization (Takechi & Kawamura, 2005). Red cones express long-wavelength-sensitive (LWS) opsins. In red cones, message for the shorter-wavelength-peaking LWS2 opsin is expressed first, at about 2 days post fertilization (dpf), comes to dominate the whole surface of larval retina, and maintains strong expression in a wide area of adult central retina. LWS1 is first seen at about 3–5 dpf in a few cones located in the ventral retina [upward looking or strike zone (Zimmermann et al., 2018)]. Dense expression in this area continues through adulthood, expands to all peripheral retina, and less densely occupies all central retina (Takechi & Kawamura, 2005). Adults robustly express message for both LWS1 and LWS2 red opsins.

The four green cone opsins (RH2-1, RH2-2, RH2-3, and RH2-4) also follow a developmental sequence. Green opsin numbering follows wavelength peaks, from shortest (RH2-1) to longest (RH2-4) (Chinen et al., 2003). As in red cone opsins, the shortest wavelength green cone opsin (RH2-1) is expressed first, at about 2 dpf, starting in nasal retina and then covering the whole larval retina. It is shortly followed by the second shortest wavelength green opsin (RH2-2), which also comes to cover the whole larval retina. RNA expression of the longer-wavelength-peaking RH2-3 or RH2-4 becomes active by 3 dpf, beginning in far nasal retina, and continuing to expand in this region through adulthood, so that in adults, all four green cone opsins are transcribed.

Blue and UV cones are thought to express only one opsin throughout development, SWS2 and SWS1, respectively. SWS1 RNA appears by 2–3 days and covers the retinal surface by 3–4 days (Takechi et al., 2003). Blue opsin (SWS2) is as robustly found as UV (SWS1) opsin by 4 dpf (Allison et al., 2010).

### A method for following *in situ* opsin signals contained within electroretinogram data

Testing the predictions of *in situ* hybridization gene expression patterns with *in situ* cone spectral measurements is desirable. This pertains not only to the identification of the *in situ* physiological peaks but also to the progress of their developmental courses. Adult zebrafish opsins *in situ* have been assessed by microspectrophotometry (MSP) (Allison et al., 2004; Cameron, 2002; Enright et al., 2015; Levine et al., 1979; Nawrocki et al., 1985; Robinson et al., 1993; Tarboush et al., 2012) and by patch recording of cones (Endeman et al., 2013). For R-cone opsins, these *in situ* measurements suggest wavelength peaks ∼15 nm longer than found in solubilized extracts of expression systems (Chinen et al., 2003). In larvae, however, there has been only one attempt to study cone opsins by MSP (Nawrocki et al., 1985), likely due to the extremely small size of larval cones.

To compare molecular findings of opsin expression patterns in zebrafish development with *in situ* physiological measures, we employ a computational approach to extract opsin peaks from electroretinogram (ERG) spectral datasets. In larval and adult isolated eyes, the massed cone response is isolated by saturating inner-retina postsynaptic glutamatergic mechanisms with L-aspartate (Sillman et al., 1969). The resulting evoked potential, called cone PIII, is modeled as arising by linear summation of voltages from four zebrafish cone types. In the model, each of these cone signals is represented by a Hill function, with a saturation voltage, semisaturation irradiance, and an opsin peak. It is the course of these opsin peaks and their quantal half-saturation sensitivities that we follow through zebrafish retinal development.

Multiple opsins, with expression patterns changing in space and time, are potentially a sensitive developmental system. For this reason, we examine the patterns in two common zebrafish background strains, the wild-type (WT) and coloration mutant *roy orbison* (roy) (D’Agati et al., 2017), and the latter, because of relative transparency, often used for imaging studies (Mumm et al., 2006). By examining two strains, the robustness of developmental patterns can be judged.

## Materials and methods

### Zebrafish

Zebrafish (*Danio rerio*) were kept in stand-alone, recirculating, Aquatic Habitats benchtop systems (Pentair Aquatic Eco-Systems, Apopka, Florida), using protocols for breeding and experimentation approved by the National Institute of Neurological Disorders and Stroke/National Institute on Deafness and Other Communication Disorders/National Center for Complementary and Integrative Health IACUC (ASP 1307, ASP 1227). Homozygous *roy orbison* (D’Agati et al., 2017) and WT larvae were spawned and eyes harvested at 5, 6, 7, and 12 dpf. For days 5–7, larvae were incubated at 28°C in 3.5-inch Petri dishes atop a heating pad. Larval medium contained 60 mg/liter sea salt, 75 *µ*l/liter 1.5% methylene blue (Sigma-Aldrich Cat. No. 03978); 5–7 dpf larvae were not fed; 12-day larvae were kept in system nursery tanks (520–650 *µ*Ω water, 28°C, pH 7.5–7.7) and fed both Larval AP100 (Pentair Aquatic Eco-Systems) and live rotifers (*Brachionus plicatilis*; Reed Mariculture, Campbell, California). Adult roy or WT were 8–18 months of age at the time of data collection. Adults were fed pulverized TetraMin flakes (Tetra GMBH, Melle, Germany) and live rotifers; they were kept in the same habitat as 12-dpf larvae.

### Isolation of larval eyes

At 5, 6, 7, or 12 dpf, larvae, captured and isolated on a glass lantern slide in a minimal volume of larval incubation water, were adsorbed onto a chip of black nitrocellulose filter paper (Millipore, 0.45 *µ*m pore, Cat. No. HABP02500, MilliporeSigma, Burlington, Massachusetts) and decapitated (without anesthesia) using a long (37 mm) insect pin (Carolina Biological Supply, Burlington, North Carolina). Using a binocular microscope (MZ12-5; Leica Microsystems, Buffalo Grove, Illinois), a longitudinal, dorsal–ventral cut through the head isolated larval eyes, which were positioned facing up, taking care not to touch the eye directly. In the recording system, larval eyes, mounted on the nitrocellulose chip, were perfused at 0.1 ml/min with minimal essential medium (MEM; Thermo Fisher Scientific, Waltham, Massachusetts, Cat. No. 11090-099, equilibrated with 95%O_2_ and 5% CO_2_) using a syringe pump (New Era 500L; Braintree Scientific, Braintree, Massachusetts) and a 28-gauge Microfil syringe needle as applicator (MF28G67; World Precision Instruments, Sarasota, Florida). The chamber consisted of an inverted lid for a 35-mm culture dish (Thermo Fisher Scientific), with a disk of 41 *µ*m nylon net filter (Millipore) covering the bottom to wick away perfusate. The Microfil applicator was positioned on the nylon mesh. 20 mm L-aspartate (Sigma-Aldrich, St Louis, Missouri), added to MEM perfusate, blocked postsynaptic, glutamatergic, photoreceptor mechanisms. Patch electrodes (3 *µ*m tip) were inserted trans-corneally to record the massed cone PIII ERG signals.

### Preparation of adult eyecups

Light-adapted adult zebrafish were decapitated with a fresh single-edged razor and the heads longitudinally hemisected between the eyes. Eyes were removed, carefully cleaned of attached tissue, and mounted upright on 5–10 mm squares of black nitrocellulose filter paper. Under a binocular microscope, the cornea, iris, and lens were removed. In the recording chamber, eyecups were perfused with MEM (as above) containing 10 mm L-aspartate at 0.3 ml/min. The perfusion applicator was placed directly in the eyecup to ensure retinal oxygenation. Broken microelectrodes (300 *µ*m tips), placed in the eyecup, recorded cone-PIII signals (Nelson & Singla, 2009).

### Spectral stimulator

Monochromatic light stimuli were obtained from a 150W OFR Xenon arc, shutter (Cat. No. LS6ZM2, 300 ms steps at 2.5–6.0 s intervals; Vincent Associates, Rochester, NY), interference filters (330-650 nm, 40 nm increments, 20 nm half-width; Chroma Technology, Bellows Falls, Vermont), metallic neutral density filters (7.5 log units, 0.5 log unit steps; Andover Corporation, Salem, New Hampshire), computer-driven filter wheels, and liquid light guide (Lambda 10-3; Sutter Instruments, Novato, California). Stimuli entered the epifluorescence port of an Olympus BX51 upright microscope (Olympus—Life Science Solutions, Waltham, Massachusetts) were redirected by a custom cold mirror cube (Chroma Technology) and were projected onto stage-mounted perfused eyes through UV-compliant objective lenses. A 10× UPlanFLN/0.3 projected stimuli onto isolated larval eyes, and a 4× UPlanSApo/0.16 projected stimuli onto adult eyecups. As calibrated in the plane of the eye (818 series calibrated photodiode; Newport Corporation, Irvine, California), the source was nearly spectrally flat from 650–370 nm, but 10× attenuated at 330 nm. All spectral model calculations are based on absolute, wavelength-specific photodiode calibrations of quanta delivered to the eye.

A background beam was projected onto the eyes through a second light path. Infrared (IR, RG780 long-pass filter, *λ* > 780 nm) served as the “neutral” background for IR visualization of eye and electrode placement using an IR camera (Teledyne QImaging, Surrey, British Columbia; Retiga-2000RV) and imaging software (Metamorph; Molecular Devices, San Jose, California). In addition to this IR background, the second light path provided red (627 nm) and blue (418 nm) backgrounds, using a dichroic interference filter, combined with either red- or blue-colored glass filters to eliminate the unused wavelength.

### Spectral stimulus protocol

The ∼20-min spectral protocol was a fixed sequence of 280, 300 ms, monochromatic light flashes of different irradiances delivered by computer using in-house software. There were 64 unique stimuli and 6 replicates. This created a set of 70, 4× averaged, ERG responses, which is called a “spectral dataset.” Wavelengths were given in the order 650, 570, 490, 410, 330, 650, 610, 530, 450, and 370 nm. Replicates at 650 nm provided an index of response stability. At each wavelength, a staircase of seven irradiances was delivered in 0.5 log unit steps, with brightness range preadjusted to cover the anticipated response range from threshold through saturation. The interval between stimuli varied between 2.5 and 6 s, with the longer intervals separating the brighter stimuli. The protocol settings for maximum irradiances in log(quanta·μm^−2^·s^−1^) at each wavelength were 7.2 (650 nm), 6.3 (610 nm), 6.4 (570 nm), 6.3 (530 nm), 6.4 (490 nm), 6.1 (450 nm), 5.7 (410 nm), 5.7 (370 nm), and 5.2 (330 nm).

### Amplification, digitization, and positioning

The microscope was positioned over the eye with a translation stage (MT-800; Sutter Instrument, Novato, California). Microelectrodes were inserted into eyes (or eyecups) with a micropositioner (MPC-385; Sutter Instrument). Microelectrode signals were amplified by 10,000 (DAM80, 0.1 Hz-1 kHz bandpass; World Precision Instruments), and digitized (2000 Hz) with an Axon instruments 1440A (Molecular Devices) using Clampex 10 software. Setting the Clampex averaging feature to retain all the elements of the average, the 280 ERGs within a single spectral dataset were captured in a single file.

### Measurement of spectral data

Spectral dataset files were imported into Origin analysis software and processed using Origin LabTalk scripts (Origin, various versions; Originlab Corporation, Northampton, Massachusetts). The four replicate waveforms at each of the 70 wavelength/brightness combinations were averaged and boxcar filtered (17 ms, one 60 Hz line frequency cycle). Peak-to-trough amplitudes were extracted during the 850 ms following stimulus onset, an interval including both the hyperpolarizing trough and repolarizing peak of the PIII response. These amplitudes were associated in Origin with the wavelength and irradiance of the stimulus, providing 70 wavelength, irradiance, and amplitude data points from each spectral dataset. Spectral datasets with unstable response amplitudes over the collection period were rejected. Nonlinear fits of models to spectral datasets used the Levenberg–Marquardt iteration algorithm provided by Origin.

### Combining and normalizing datasets from many eyes

One to three datasets were acquired from each eye, and the datasets from 6–16 eyes were combined into single large datasets for each strain, age, and background condition. These are “treatment-level” datasets, where “treatment” refers to common strain, age, background illumination, and stimulation protocol. These large, treatment-level datasets contained between 61 and 3220 stimulus-response data points. When fitting spectral models to datasets, there is a statistical advantage to combining datasets from all eyes in the same treatment group, as the standard error (s.e.) on fit parameters shrinks, though, because of variability among eyes, *r*^2^ is reduced. In a final summary of mean properties for larval cones, treatment-level datasets from all larval ages were combined, and multi-age treatment-level datasets of up to 6440 points were fit.

The largest source of variance within combined spectral datasets is variance in peak amplitudes among individual eyes. To eliminate this variance, we included the option of normalizing the individual spectral datasets to the peak amplitude within, before combining them into the large, treatment-level dataset. In this way, all datasets are given equal weight in the fitting process. In comparing spectral sensitivity, or irradiance response fits between different treatment-level datasets, the means of the peak amplitudes of spectral datasets contained within them were first compared. If these means did not differ (*t*-test, *α* = 0.05), then individual spectral datasets were normalized before fitting the spectral functions to be compared. This condition was met for all spectral plots arising from present treatment-level datasets, and so it is the fits to normalized treatment-level datasets that are compared herein.

Opsin spectral peaks are independent of cone PIII amplitude. Therefore, large, normalized, treatment-level datasets were used for computing opsin peaks and SEs. In this way, fit error is reduced. When determining differences in fit opsin peaks, either the ‘Analysis of Variance from Summary Data’ ANOVA web calculator was used (http://statpages.info/anova1sm.html), or, similarly for *t*-tests, the Graphpad web calculator was used (https://www.graphpad.com/quickcalcs/ttest1/?Format=SEM). In these statistical comparisons, “*n*” is the number of voltage responses to stimuli in the treatment-level dataset.

## Results

### A model to compute opsin peaks from cone PIII spectral data

#### Cone opsin nomogram functions

As represented on the normalized energy axis (*λ*_max_/*λ*, where *λ*_max_ is the peak absorbance wavelength and *λ* is the absorbance at any wavelength), cone opsin absorbance functions are similar in shape (Dartnall, 1953). In effect translating a single cone opsin shape along a reciprocal wavelength axis closely fits opsin absorbance spectra for a variety of opsin wavelength peaks. This nomogram absorbance function, *A*(*λ*_max_, *λ*), is typically represented by a polynomial. In the present model, the order 8 polynomial function of Hughes et al. (Hughes et al., 1998) represents zebrafish red, green, and blue opsins (r, g, b). The order 8 polynomial of Palacios et al. is used for zebrafish UV opsin (u), as UV opsin is narrower in spectral shape, and does not conform to the longer-wavelength cone opsin nomogram. Both nomograms are based in the suction-electrode photocurrents of isolated cones in giant danio and represent opsin photocurrent data from 277–697 nm (Palacios et al., 1996). The polynomial coefficients are reproduced in Table 1.

**Table 1.** Polynomial coefficients for normalized cone opsin absorbance nomograms, *A*(*λ*_max_, *λ*)

Coefficient	R, G, B cones (Hughes et al., 1998)	UV cones (Palacios et al., 1996)
*C*_0_	−93.262	−48139.554
*C*_1_	617.458	309007.504
*C*_2_	−2815.735	−770875.542
*C*_3_	7339.277	834365.023
*C*_4_	−10714.545	−57657.482
*C*_5_	9048.982	−815689.388
*C*_6_	−4410.667	871189.984
*C*_7_	1154.235	−389189.665
*C*_8_	−125.738	66989.122

**Fig. 1.**
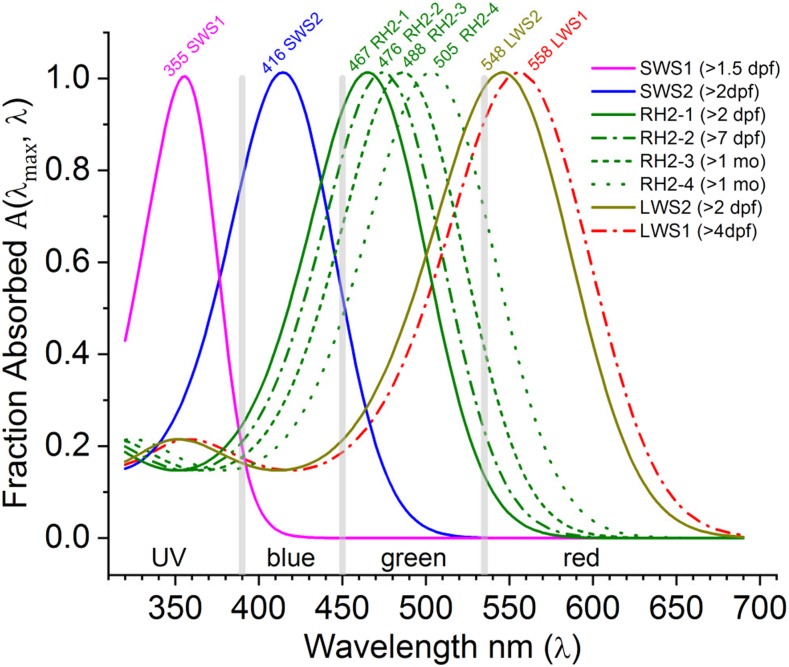
Opsin nomogram function *A*(*λ*_max_, *λ*) fills out absorbance shapes for the eight zebrafish cone opsins based on peaks reported by Chinen et al. (2003). The legend lists the developmental timing for each opsin mRNA expression (Takechi & Kawamura, 2005). For green (RH2) and red (LWS) opsins, there is a predicted progression toward longer wavelengths with development. Solid lines are the early larval expression patterns. Dashed and dotted lines represent opsin mRNAs expressed later in larval, juvenile, and adult development. The nomograms are order 8 polynomials (Hughes et al., 1998; Palacios et al., 1996), with literature coefficients listed in Table 1. Based on these opsin peaks, the spectrum was divided into UV, blue, green, and red wavebands (vertical gray bars), in which to search for physiological opsin peaks for U-cones (SWS1 opsin), B-cones (SWS2 opsin), G-cones (RH2 opsin), and R-cones (LWS1, LWS2 opsins).

In Fig. 1, the *A*(*λ*_max_, *λ*) function is used to infer the spectral shapes of eight zebrafish cone opsins using the published absorbance peaks in solution (Chinen et al., 2003). Later developmental studies of mRNA expression predict a physiological shift in opsin sensitivities from shorter wavelengths (solid curves, larvae) toward longer wavelengths (dashed curves, adults) for green and red opsins (RH2 and LWS, respectively) (Takechi & Kawamura, 2005). The goal herein is to test these predictions with a physiological measure.

#### Wavelength-dependent Hill function for each cone

The intensity response functions of cones are well represented by Hill functions [eqn. (1)], also called Naka–Rushton functions (Naka & Rushton, 1966), with two parameters, a maximal response amplitude (*V*_max_) and a semisaturation value for stimulus irradiance (*k*).1
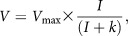
*V* is the response amplitude for a stimulus of irradiance *I*. To enter wavelength dependence (*λ*) into eqn. (1), *k* is divided by the cone opsin nomogram absorbance, *A*(*λ*_max_, *λ*) (Fig. 1). This function is normalized to the absorbance peak, so that *k* now refers to semisaturation irradiance at the wavelength of opsin peak absorbance (*λ*_max_). Eqn. (2) is the wavelength-dependent Hill function.2
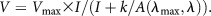


In zebrafish, cone-PIII ERG signals are assumed to be the sum of signals from multiple cone types, each expressing an opsin with a different absorbance peak (*λ*_max_), each with a separate saturation amplitude (*V*_max_), and each with a separate semisaturation irradiance (*k*). Adult zebrafish are thought to have four cone types (Engstrom, 1960), double cones, with the principal type expressing red opsin (r), and the accessory member green opsin (g), long singles, with blue opsin (b), and short singles, with UV opsin (u) (Robinson et al., 1993). Larval zebrafish lack double cones; nonetheless, the evidence also favors four cone types, with the red and green cones being separately represented by long single types, which combine into double cones later in development (Allison et al., 2010). The cone PIII amplitude representation as a four-cone signal summation appears in eqn. (3), the “spectral model.”3
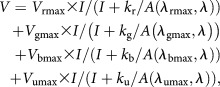
There are 12 fit parameters for the PIII spectral datasets; the saturation voltages for red, green, blue, and UV cones (*V*_rmax_, *V*_gmax_, *V*_bmax_, and *V*_umax_); the semisaturation irradiances for these cones (*k*_r_, *k*_g_, *k*_b_, and *k*_u_); and the peak absorbance wavelengths for the physiologically active opsins (*λ*_rmax_, *λ*_gmax_, *λ*_bmax_, and *λ*_umax_). A fit to all 12 parameters was not necessarily found in every dataset, for example, because signal strength of a particular cone type, or cone types, might be too weak, or that no clear opsin peak could be found within the waveband boundaries examined. In such cases, the semisaturation irradiances (*k*) and wavelength peaks (*λ*_max_) did not converge on a value within waveband boundaries. If *k* or *λ*_max_ iterations reached a boundary value for the fit, it was a sign of a failure to fit. In such cases, the parameter was set to a fixed value, typical of that cone type and the dataset refit.

Because of the extended range, semisaturation irradiances are both given and fit as log units (log *k*_r_, log *k*_g_, log *k*_b_, and log *k*_u_), in units of log(quanta·μm^−2^·s^−1^). The log *k* boundaries were 1–6 log(quanta·μm^−2^·s^−1^). Fit values outside this range were excluded as nonphysiological, a fixed value more typical of that cone type was substituted, and the model fit was recomputed. These fixed values were 4.5 (red cones), 4.0 (green cones), 3.5 (blue cones), and 3.0 (UV cones), as previously reported (Nelson & Singla, 2009). For the boundaries on opsin *λ*_max_, the spectrum was split into four nonoverlapping wavebands, one for each opsin (Fig. 1, vertical gray bars). Based on the literature expectations for adult zebrafish (Allison et al., 2004; Cameron, 2002; Chinen et al., 2003; Endeman et al., 2013; Enright et al., 2015; Levine et al., 1979; Nawrocki et al., 1985; Robinson et al., 1993; Tarboush et al., 2012), these wavebands were set at 535–630 nm (R-cones), 450–534 nm (G-cones), 390–449 nm (B-cones), and 330–389 nm (U-cones). If an opsin peak was not found within waveband boundaries, a fixed value was substituted, and the calculation repeated. These fixed values are 555 nm (R-cones), 475 nm (G-cones), 415 nm (B-cones), and 362 nm (U-cones), as assessed from literature. *V*_max_ boundary values were (±500 *µ*V), and in practice, not reached.

### Sample cone-PIII spectral datasets

**Fig. 2.**
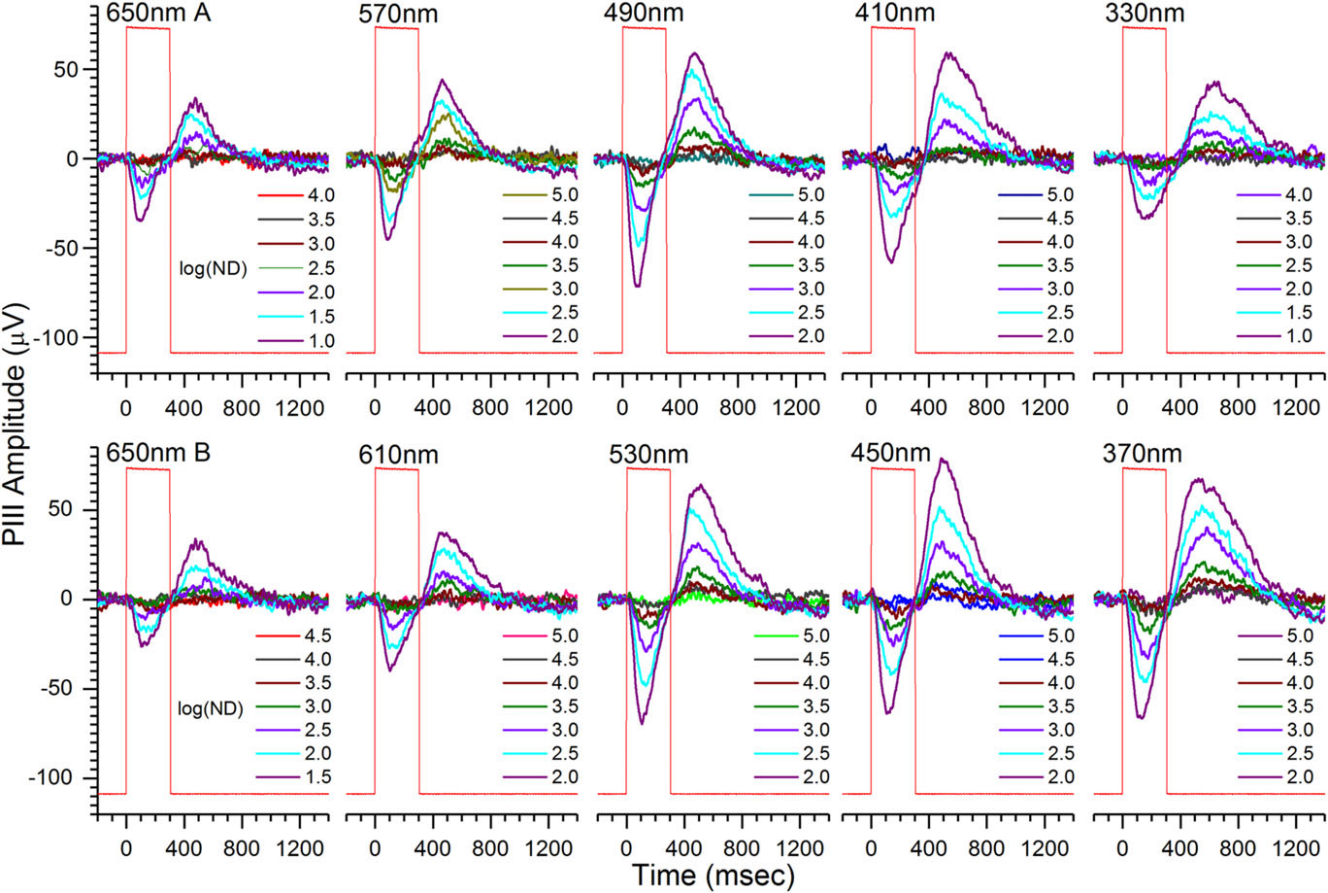
Cone ERG PIII light-response waveforms contained in an individual spectral dataset from an isolated, perfused, 6-dpf roy larval eye. Cone PIII signals consist of transient vitreal negativity during stimulation, followed by vitreal positivity, or rebound, after light offset. The peak-to-peak amplitudes during the 850 ms following stimulation are used in extracting R-cone, G-cone, B-cone, and U-cone signal saturation voltages, semisaturation irradiances, and wavelength peaks. Stimulus wavelengths appear above each nested, seven-step irradiance-response series. The time-order of brightness is dim to bright at each wavelength, with wavelengths presented in temporal order left to right and top to bottom. Each waveform is an average of 4. There are 70 mean signals in each dataset. Larval PIII responses are obtained in 20 mm aspartate MEM on an IR (*λ* > 780 nm) background. Rectangular steps (red) are photocell responses to the light stimulus. The log of neutral density attenuating the monochromatic beam, log(ND), is given in each legend. Greater log(ND) corresponds to dimmer stimulation. Less log(ND) stimulus attenuation is used at 650 nm to compensate for the low absorbance of all opsins at this wavelength, and less log(ND) attenuation is used at 330 nm, to compensate for the 10× attenuation of this wavelength by the microscope objective.

A single, 70-waveform, spectral dataset for a 6 dpf roy larva is shown in Fig. 2. The roy phenotype is a stripe-less, semitransparent coloration mutant with black rather than yellow iris and lacking reflective iridophores (D’Agati et al., 2017). Cone-PIII responses consist of a transient-ON vitreal negativity, peaking *during* the 300 ms stimulus, with a vitreal-positive OFF peak of about equivalent amplitude *following* stimulus offset. Shorter wavelengths evoke greater amplitudes (Fig. 2). In the modeling of eqn. (3), this results from the poor photon absorbance of any opsin for wavelengths longer than 570 nm, and from the addition of signals from green, blue, and UV cone types with progressively shorter wavelength stimuli. The dataset was obtained on an IR (*λ* > 780 nm) background.

**Fig. 3.**
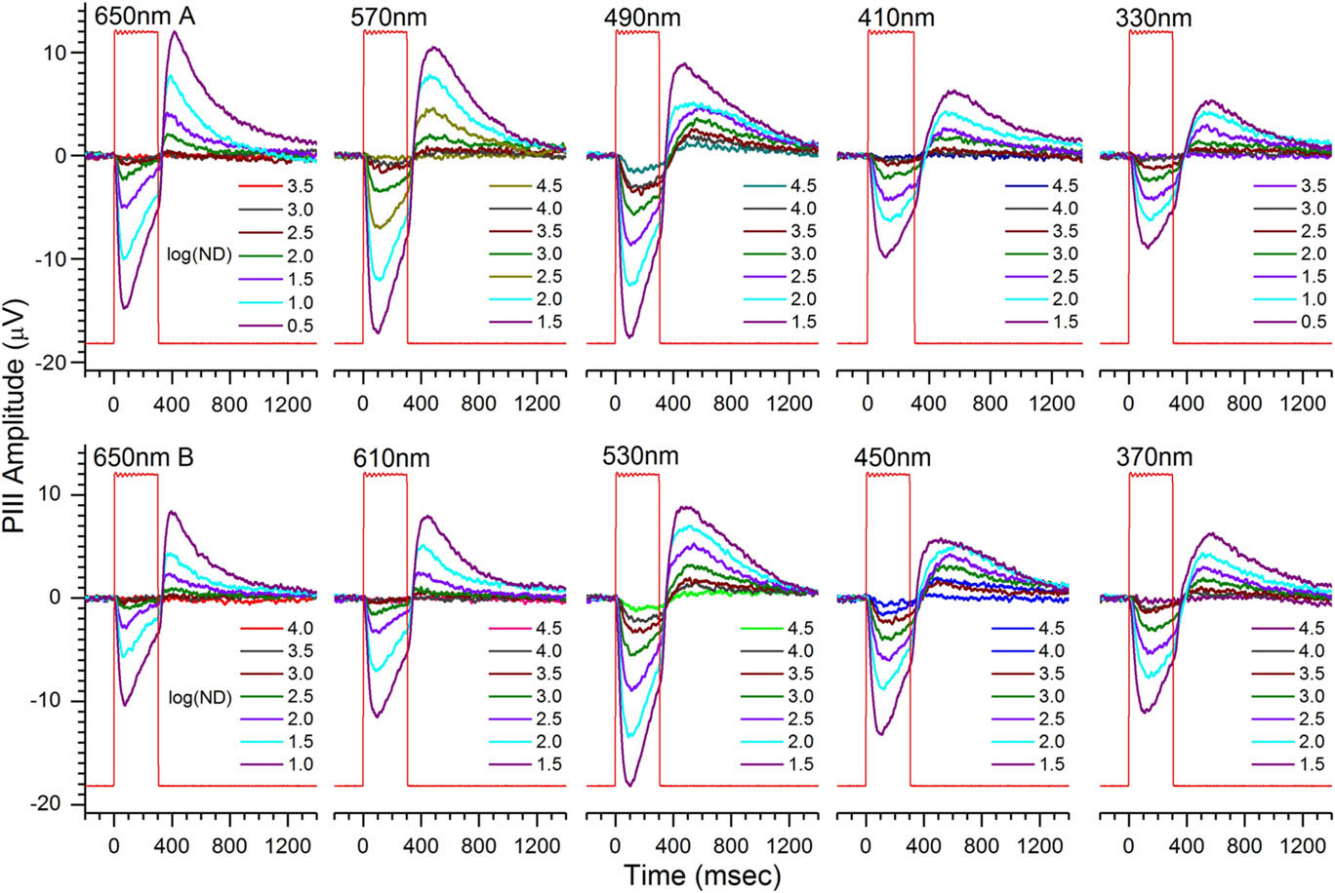
Individual spectral dataset from an isolated, perfused, adult roy eyecup. Cone PIII signals consist of transient-sustained vitreal negativity during stimulation, followed by vitreal positivity after light offset. The peak-to-peak amplitudes of these waveforms are used to model properties of the component cone signals. Conditions are the same as described in Fig. 2, except, the perfusate is 10 mM aspartate MEM, and the background illumination is red (627 nm).

Fig. 3 is a single spectral dataset from an adult roy eyecup obtained on a red (627 nm) background. Red and blue backgrounds are used to search for multiple opsin peaks within a waveband through selective desensitization. As compared with larval waveforms, the vitreal negativity of these red-adapted adult PIII signals is more sustained during light stimulation, and the depolarizing rebound after stimulus offset is proportionally not as large. In adults, overall amplitude is not as large as with larvae, a feature caused by the short-circuiting effect of an open, perfused eyecup preparation, with cornea and lens removed. The largest amplitude signals are seen with mid-spectral stimulation, where photons are captured by both red and green cones. Waveforms at 650 nm appear significantly quicker to peak, particularly at offset. The 627 nm red background selectively light-adapts red cones, accelerating red cone light-signal kinetics, raising the semisaturation level, but not affecting the amplitude of response.

In Figs. 2 and 3, because of aspartate treatment, there is no b-wave. In untreated retinas, b-wave peaks, unlike the cone-PIII OFF peak, would occur during the 300-ms stimulus, not after it (Nelson & Singla, 2009).

### Modeling larval irradiance response data

**Fig. 4.**
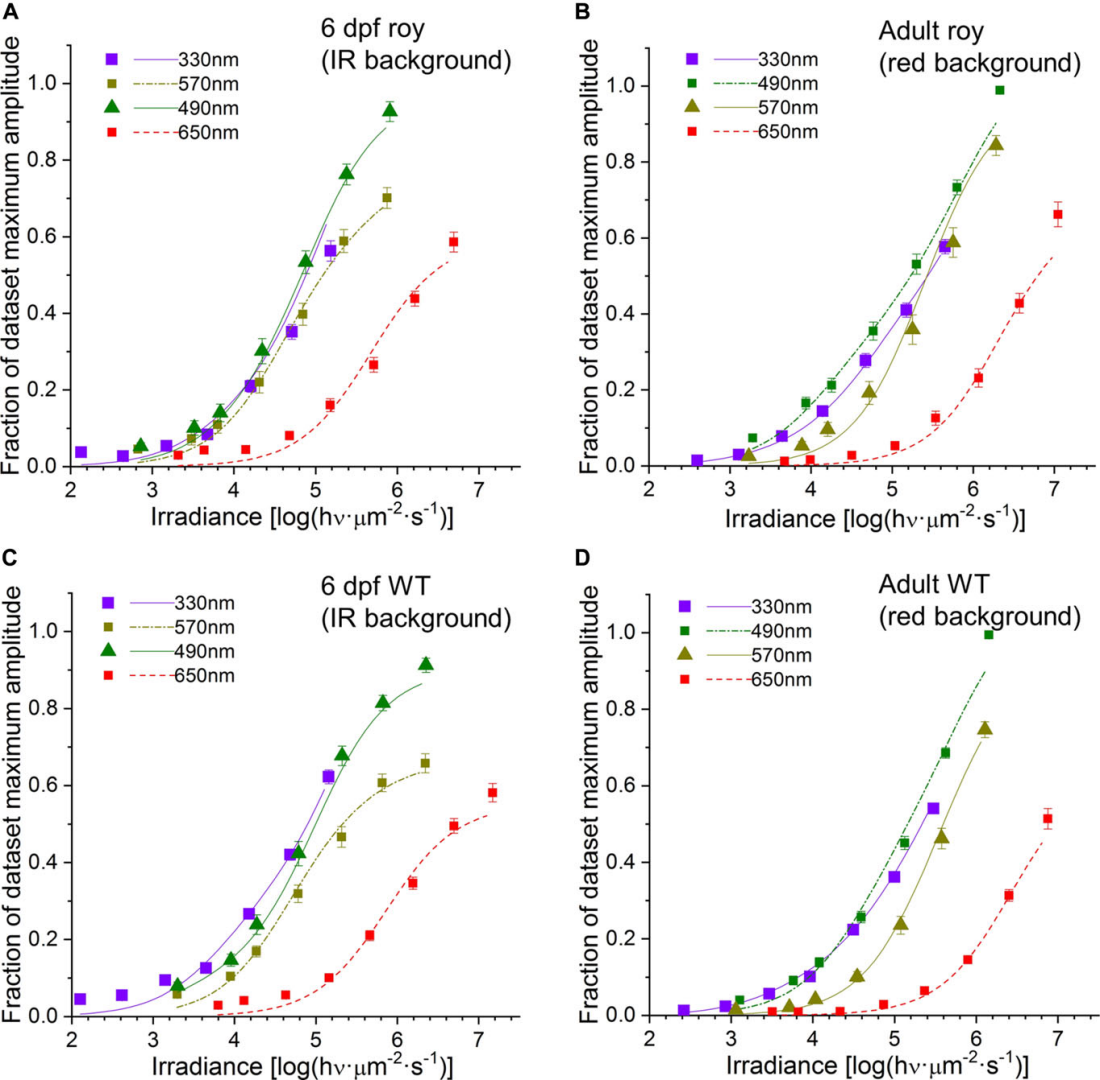
Spectral model [eqn. (3)] provides good fits to normalized, treatment-level cone PIII datasets combined from many eyes, regardless of age, strain, or background illumination. (**A**) Irradiance-amplitude plots at four wavelengths from a 6-dpf roy, 15-eye, 1610-point, normalized treatment-level dataset (IR background, *λ* > 780 nm). (**B**) Irradiance-amplitude plots for an adult roy, six-eye, 980-point, normalized treatment-level dataset (red, 627 nm background). (**C**) Irradiance-amplitude plots from a 6-dpf WT, 10-eye, 1858-point, normalized treatment-level dataset (IR background). (**D**) Irradiance-amplitude plots for an adult WT, 11-eye, 1258-point, normalized treatment level dataset (red background). Points are means ± s.e., with N for each point equal to ∼1/10th the points in each dataset, except for the 650 nm points, ∼2/10th the number of points. Curves are eqn. (3) model fits. Parameters of the model fits in A, B, C, and D appear in Fig. 5A. The semisaturation values for model curves differ from cone semisaturation values at absorbance maxima (log *k*) because model curves are composed of signals from many cones, most at wavelengths other than peak absorbance. Note the leftward shift of 330 nm curves in respect to 490 nm curves when comparing roy and WT strains.

The spectral model [eqn. (3)] fits continuous, irradiance response curves to cone-PIII amplitudes at seven irradiances and nine wavelengths. The individual curves (Fig. 4A–4D), and their semisaturations, each reflect the summation of signals from four cones, each stimulated by a single wavelength, whereas eqn. (3) log *k* semisaturations are for individual cones, each fit at the absorbance maximum. The log *k* semisaturations are inferred by the model and cannot be read directly from the plots of Fig. 4.

**Fig. 5.**
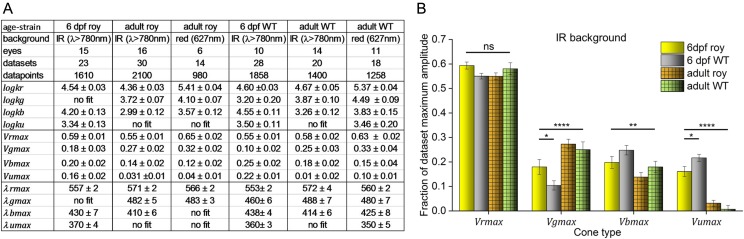
Cone-type signal amplitudes differ between roy and WT and between adults and larvae. (**A**) Parameter names are the same as in the Spectral model [eqn. (3)]. Fit values are means ± s.e. In each column, the model is fit to a treatment-level dataset composed of individual datasets from multiple eyes, each normalized to its own maximal response. The irradiance response curves corresponding to four of these six model fits appear in Fig. 4. (**B**) WT and roy, larval and adult, cone saturation amplitudes are compared on IR backgrounds (*λ* > 780 nm). G-cone saturation amplitudes are greater in adults than in larvae, while B- and U-cone signals are less (for ANOVAs, see text). G-cone saturation amplitudes are greater in 6-dpf roy than in WT larvae, while U-cone signals are less (*t*-tests, see text). *V*_rmax_, *V*_gmax_, *V*_bmax_, and *V*_umax_ are saturation amplitudes of R-, G-, B-, and U-cones as a fraction of individual dataset maxima [values from (**A**)]. Statistical comparisons are annotated in Graphpad Prism convention, where * means *P* < 0.05; **, *P* < 0.01; ***, *P* < 0.001; ****, *P* < 0.0001; *****, *P* < 0.00001; ns, not significant.

A comparison of response amplitude points with modeled irradiance-amplitude curves is an index of model success. In Fig. 4A, a treatment-level dataset is compiled from 23 datasets (each like Fig. 2), from 6-dpf roy *in vitro* eyes, in the presence of aspartate medium and an IR background. Each spectral dataset within the treatment-level dataset was first normalized to the maximal amplitude in the dataset before compiling. This treatment-level dataset was obtained from 15 eyes, amassing 1610 wavelength-irradiance-amplitude points. The four irradiance-amplitude curves (at 330, 490, 570, and 650 nm) are model generated, using the parameter fits (Fig. 5A, 6 dpf roy) to the full, 9-wavelength, 7-irradiance, multi-eye treatment-level dataset. The points are the means and s.e.s from within the treatment-level dataset, showing the measured amplitude *vs.* irradiance values at the four wavelengths. Model curves closely follow these data points. This pan-spectral model [eqn. (3)] accurately captures larval irradiance response functions at multiple wavelengths in larval datasets.

Larval irradiance response curves and data points from a WT, 6-dpf, treatment-level, 1858-point dataset are shown in Fig. 4C. Parameter fits are shown in Fig. 5A (6 dpf WT). The 330 nm curve lies to the right of the 490 nm curve for the 6-dpf roy dataset but lies to the left of the 490 nm curve for the WT dataset, suggesting a spectral difference between strains.

### Modeling adult irradiance response data

In Fig. 4B, a treatment-level dataset, compiled from 14 normalized cone-PIII datasets (like Fig. 3), taken on red-adapted (627 nm) adult, Aspartate-treated, roy eyecups, is fit by the model, and irradiance-amplitude curves are generated from model parameters (given in Fig. 5A). The points are mean amplitudes (±s.e.) at each irradiance and wavelength taken from within the treatment-level dataset. Model curves closely follow points at each of the four wavelengths. The pan-spectral model [eqn. (3)] well represents irradiance-amplitude plots at multiple wavelengths in adult eyecups, even under conditions of chromatic adaptation. In Fig. 4D, irradiance response curves are fit for a WT adult treatment-level dataset (fit parameters in Fig. 5A) also aspartate-treated, and under conditions of red (627 nm) background. Like 6-dpf roy eyes, in adult roy eyecups, the 330 nm irradiance-amplitude curve lies farther to the right of the 490 nm curve (Fig. 4B) than in WT (Fig. 4D), suggesting a developmentally persistent spectral distinction between strains.

### Modeling chromatic backgrounds

The spectral model finds that the red cone semisaturation value (log *k*_r_ = 5.41, Fig. 5A, adult roy, red) is increased by one log unit as compared with IR semisaturation (log *k*_r_ = 4.36, Fig. 5A, adult roy, IR, *t*-test, *t* = 20.3, *df* = 3078, *P* < 0.0001), and similarly for WT, by about 0.7 log units, from log *k*_r_ = 4.67 on IR background to log *k*_r_ = 5.37 on red background (Fig. 5A, *t*-test, *t* = 10.8, *df* = 2656, *P* < 0.0001). Adult cone saturation amplitudes are not reduced by 627 nm backgrounds, but either remain the same or increase (Fig. 5A). The model implies that the red background increases semisaturation in red cones, without reducing maximum signal voltage. The ability of cones to desensitize without losing response amplitude was originally reported in mudpuppy cones (Normann & Werblin, 1974), also known to be red cones (Fain, 1975).

### Opsin peaks

The model infers wavelength maxima for cone signals within opsin wavebands (Fig. 5A). For 6-dpf roy larvae, peaks are found for three of the four cone signals: R-cone, *λ*_rmax_, 557 ± 2 nm; B-cone, *λ*_bmax_, 430 ± 7 nm; and U-cone, *λ*_umax_, 370 ± 4 nm. For 6-dpf WT larvae, opsin peaks were fit in all four wavebands: R-cone, *λ*_rmax_, 553 ± 2 nm; G-cone, *λ*_gmax_, 460 ± 6 nm; B-cone, *λ*_bmax_, 438 ± 4 nm; and U-cone *λ*_umax_, 360 ± 3 nm. In the adult roy strain on IR backgrounds, the model-fit opsin peaks are as follows: R-cone, *λ*_rmax_, 571 ± 2 nm; G-cone, *λ*_gmax_, 482 ± 5 nm; and B-cone, *λ*_bmax_, 410 ± 6 nm. In adult WT on IR backgrounds, fits were obtained for R-, G-, and B-cones: *λ*_rmax_, 572 ± 4 nm; *λ*_gmax_, 488 ± 7; and *λ*_bmax_, 414 ± 6 nm. In adult WT on red background, a U-cone fit was found: *λ*_umax_, 350 ± 5 nm.

### Signal strengths of cone types in larvae and adults

Larvae differ from adults both in cone mosaic pattern and in patterns of opsin mRNA expression (Allison et al., 2010; Takechi & Kawamura, 2005). Four of the parameters fit by the spectral model [eqn. (3)] are the cone-PIII saturation amplitudes for R-, G-, B-, and U-cones. These are indexes of the physiological signal levels for these cone types. A developmental change in amplitude distribution among types is apparent (Fig. 5B). R-cone signal amplitudes (*V*_rmax_) are the largest and do not differ significantly among roy and WT larvae and adults [ANOVA, *F*(3, 8964) = 1.98, *P* = 0.1153, 45 eyes]. G-cone amplitudes are significantly larger in adults than in larvae [ANOVA, *F*(3, 8964) = 10.40, *P* < 0.00001, 45 eyes]. On the other hand, B-cone signals are larger in larvae than in adults [ANOVA, *F*(3, 8964) = 5.25, *P* < 0.01, 45 eyes]. UV cone amplitudes are distinctly reduced in adults as compared with larvae [ANOVA, *F*(3, 8964) = 42.19, *P* < 0.00001, 45 eyes]. Thus, the distribution of physiological amplitudes among G-, B-, and U-cones favors shorter-wavelength B- and U-cones in the larval stage but mid-wavelength G cones in the adult stage.

At the larval stage, roy and WT strains differ in cone saturation amplitudes (Fig. 5B). The G-cone saturation amplitude in 6-dpf roy larvae is larger than in WT larvae (*t*-test, *df* = 3466, *t* = 2.2, *P* < 0.05), while the U-cone amplitude is significantly smaller (*t*-test, *df* = 3466, *t* = 2.3, *P* < 0.05).

### Larval spectral sensitivity

**Fig. 6.**
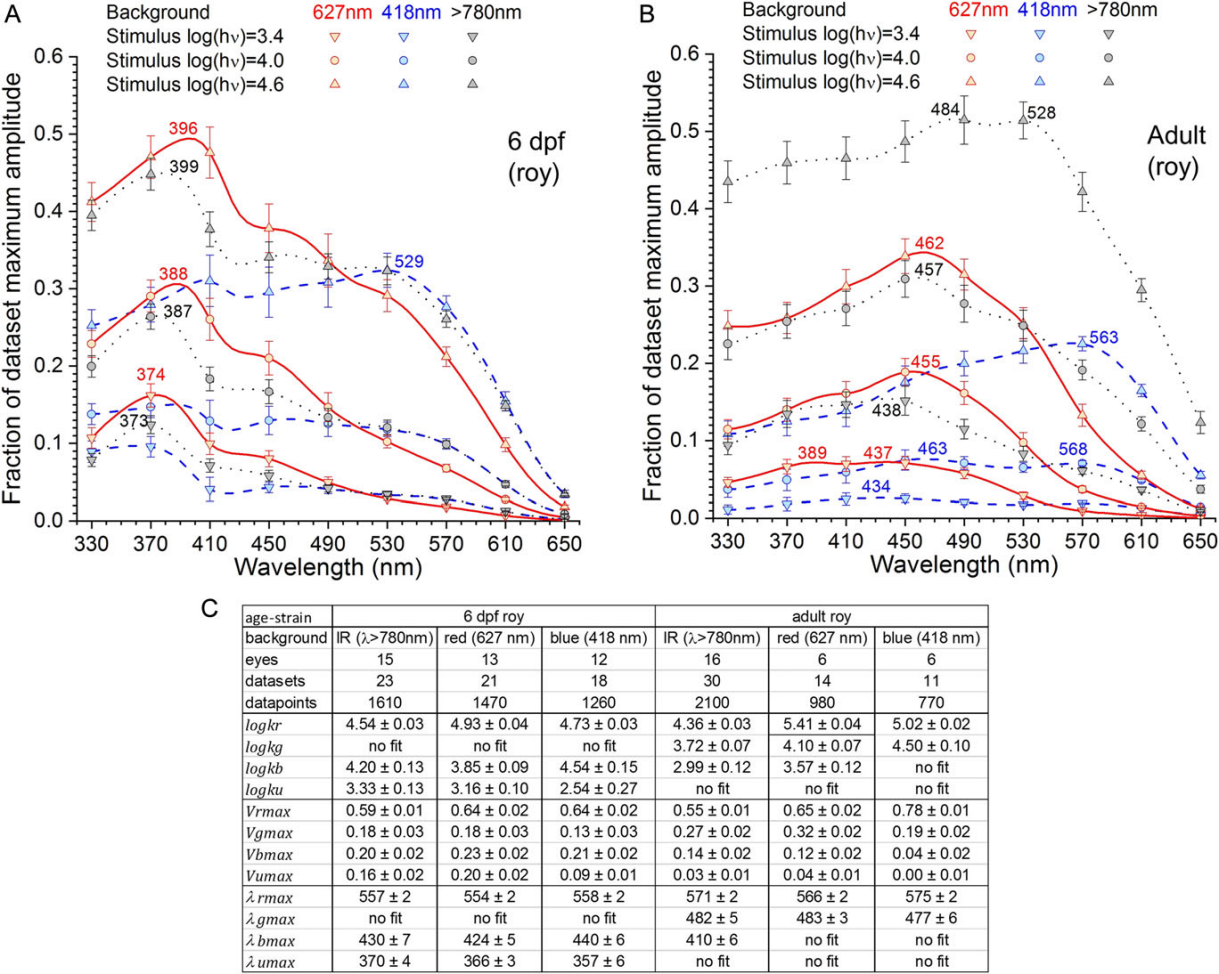
Model-generated cone-PIII spectral curves for roy larvae and adults on IR (*λ* > 780 nm), red (627 nm) and blue (418 nm) backgrounds. (**A**, **B**) For each background, model-fit, amplitude- *vs.*-wavelength curves are plotted for three constant quantal stimulus levels. These stimuli are 3.4 log(quanta·μm^−2^·s^−1^) [log(*hν*) = 3.40, dim], 4.0 log(quanta·μm^−2^·s^−1^) [log(*hν*) = 4.00, intermediate], and 4.6 log(quanta·μm^−2^·s^−1^) [log(*hν*) = 4.60, bright]. (**A**) In 6 dpf larvae, there are prominent U-cone spectral peaks (373, 374 nm) for the dim-stimulus (lowest amplitude) on red or IR backgrounds. The U-cone peaks shift to longer wavelengths (396–399 nm) with bright stimuli. R-cone signals occupy a long-wavelength spectral limb between 570 and 650 nm that becomes prominent with bright stimulation. With the blue background and bright stimuli, the long-wavelength limb develops a peak (529 nm). (**B**) The dim-stimulus, red or IR background, adult spectral peaks are in the blue (437, 438 nm). These peaks shift to longer wavelengths with bright stimuli (462, 484 nm). On the blue background, the dim-stimulus peak is 434 nm but shifts to long wavelengths (563 nm) with bright stimulation. (**C**) The spectral-model fit parameters for larvae and adults provide cone mechanisms underlying the shifting spectral shapes. For larvae, the lower semisaturation of UV cones as compared with red cones provides a UV spectral advantage for dim stimuli, but the greater saturation amplitude of red cones provides a long-wavelength spectral advantage for bright stimuli. In roy larvae, for each background condition, R-cone (*λ*_rmax_), B-cone (*λ*_bmax_), and U-cone (*λ*_umax_), but not G-cone (*λ*_gmax_), sensitivity peaks can be fit. These cone peaks differ from the peaks in the overall spectral curve in which they are embedded. The R-cone (*λ*_rmax_) peaks (554–558 nm) shift little with background wavelength [ANOVA, *F*(2, 4437) = 1.03, *P* = 0.357]. In roy adults, unlike larvae, no peaks are found for B-cones or U-cones, but peaks are fit for G-cones (*λ*_gmax_, 477–482 nm). R-cone peaks (*λ*_rmax_, 566–575 nm) shift with chromatic background (red, 566; blue 575 nm; *t*-test, *t* = 3.05, *df* = 1748, *P* < 0.01), suggesting two long-wavelength cones, R1 and R2, within the red waveband (535–610 nm), differentially sensitive to chromatic adaptation.

The spectral model [eqn. (3)] generates spectral curves from treatment-level datasets. In Fig. 6A, cone-PIII amplitudes are calculated in response to constant-quantal stimuli across wavelength for 6-dpf roy eyes. Curves are shown for three different constant quantal stimulation levels and three different chromatic backgrounds. The three constant quantal levels are dim [log(*hν*) = 3.40], intermediate [log(*hν*) = 4.00], and bright [log(*hν*) = 4.60], where *hν* is a shorthand for quanta·μm^−2^·s^−1^. A set of these three curves are each calculated for IR (*λ* > 780 nm), red (627 nm), and blue (418 nm) backgrounds. Each background is represented by a normalized, treatment-level dataset. The means of spectral dataset maxima within these treatment level datasets were 110.5 ± 12.1 (*n* = 23) for the IR background, 90.0 ± 12.0 *µ*V (*n* = 21) for the red background, and 73.1 ± 13.1 *µ*V (*n* = 18) for the blue background. These maximal amplitudes were not significantly different [ANOVA, *F*(2, 59) = 2.28, *P* = 0.11]. As peak dataset amplitudes were not significantly affected by background, little information is lost through amplitude normalization.

Zebrafish spectral sensitivity shapes are not fixed but depend both on background conditions and on the brightness of stimulation. The shapes of these curves can be understood in terms of the underlying fit parameters for each of the four cones. In the dim-irradiance spectral curves [log(*hν*) = 3.40], the larval spectral peaks for IR and red backgrounds are in the UV (373 and 374 nm, Fig. 6A). For the bright-irradiance spectral curves [log(*hν*) = 4.60], the blue-background spectral peak shifts to long wavelengths (529 nm, Fig. 6A), and, on IR or red backgrounds, a long-wavelength sensitivity limb (570–650 nm), although not including a spectral peak, becomes prominent (Fig. 6A). The increase in long-wavelength sensitivity with brighter spectral stimulation finds explanation in the different semisaturation values of U-cones (log *k*_u_) and R-cones (log *k*_r_). On the red background, semisaturation for U-cones is 1.77 log units less than R-cones, and on the IR background, the U-cone sensitivity advantage is 1.21 log units (Fig. 6C). Even though the maximal amplitude of the U-cone signal (*V*_umax_) is much less than the R-cone signal (*V*_rmax_) on any background (Fig. 6C), lower irradiance semisaturation allows U-cones to dominate the dim-irradiance spectral curves.

The long-wavelength spectral limb shifts toward shorter wavelengths on red backgrounds as compared with IR or blue backgrounds (Fig. 6A). One explanation is that a single R-cone type containing one or more R-cone opsins is selectively desensitized by red backgrounds, producing a spectral shift toward the shorter-wavelength G-cones. The second explanation is that LWS1 and LWS2, the two R-cone opsins, reside in separate R1 and R2 cones, with the longer wavelength R1 cone (LWS1 opsin) being selectively desensitized by the 627 nm red background as compared with the shorter-wavelength R2 cone (LWS2 opsin). The third explanation is a combination of the first and second.

The spectral model [eqn. (3)] separately infers wavelength maxima for cone types. Comparing the fit long-wavelength opsin peaks on red and blue backgrounds, the calculated R-opsin peak on a red background (554.0 ± 1.7, *n* = 1470, Fig. 6C) does not differ from the R opsin peak extracted on a blue background (557.6 ± 1.9 nm, *n* = 1260) with *P* = 0.16 (*t*-test). This indicates that the spectral shift toward shorter wavelengths on the red background as compared with IR or blue backgrounds is not significantly due to selective chromatic adaptation of R1 as compared with R2 cones. In 6-dpf larvae, there is mainly one R-cone with significant signals, the shorter-wavelength R2 cone (with LWS2 opsin). R2 cone desensitization, as compared with less desensitized G cones, produces most of the red-background shift to shorter wavelengths.

The roy larval B-cone peaks (*λ*_bmax_, Fig. 6C, 6 dpf) were 424.2 ± 4.7 nm (red background, *n* = 1470) and 439.8 ± 5.7 nm (blue background, *n* = 1260). These differed significantly (*t*-test, *P* < 0.05), possibly suggesting multiple cones (B1, B2) in the larval blue waveband (390–449 nm). The U-cone peaks (*λ*_umax_, Fig. 6C, 6 dpf) were 365.9 ± 2.9 nm (red background, *n* = 1470) and 356.6 ± 6.4 nm (blue background, *n* = 1260). These did not differ (*t*-test, *P* = 0.16). Despite differences in spectral shape seen with IR, red, and blue backgrounds (Fig. 6A), the model [eqn. (3)] concludes that the underlying larval R- and U-cones are mainly of a single opsin type but opens a question about B-cone opsin singularity in the blue waveband.

### Adult spectral sensitivity

In the spectral curves of Fig. 6B, cone-PIII amplitudes are modeled for constant quantal stimulation across wavelengths in adult roy perfused eyecups. Spectral shapes at three different irradiance levels (dim, intermediate, and bright) and three chromatic backgrounds (IR, *λ* > 780 nm; red, 627 nm; and blue, 418 nm) are illustrated. Thirty IR, 14 red, and 11 blue datasets were normalized before compiling into three large treatment-level datasets for IR, red, and blue background illumination. The mean of maximum dataset amplitudes was 22.5 ± 3.1 *µ*V for IR background, 13.1 ± 1.0 *µ*V for red background, and 15.8 ± 1.2 *µ*V for blue background. These values were not significantly different [ANOVA, *F*(2, 53) = 2.96, *P* = 0.060]. Since amplitudes did not differ significantly among backgrounds, comparison of spectral shapes on normalized datasets does not discard significant information.

In adult eyecups, as in perfused, isolated larval eyes, spectral shapes depend on background conditions and on brightness of stimulation. In the roy adult, dim-irradiance spectral curves [log(*hν*) = 3.40] peak in the blue (438 nm, IR background; 437 nm red background; 434 nm, blue background; Fig. 6B), rather than in the UV (<400 nm, Fig. 6A) as seen in larvae. In the bright spectral curves [log(*hν*) = 4.60], the blue-background spectral peak shifts to long wavelengths (563 nm, Fig. 6B). On the red background, the bright-stimulus amplitude peak remains mid-spectral but shifts to longer wavelengths (462 nm, Fig. 6B). The bright-stimulus curve with IR background is intermediate between the curves on red or blue backgrounds, showing spectral peaks at both 484 and 528 nm. The shift to longer-wavelength sensitivity with brighter spectral stimulation comes about in adults because, on any background, G-cone semisaturation (log *k*_g_) occurs with dimmer stimuli than required for R-cone semisaturation (log *k*_r_) (*t*-tests: IR background, *t* = 8.4, *df* = 4198, *P* < 0.0001; red background, *t* = 16.2, *df* = 1958, *P* = <0.0001; blue background, *t* = 4.8, *df* = 1538, *P* < 0.0001; log *k*_g_ and log *k*_r_ comparisons from Fig. 6C). On all backgrounds, the R-cone signal, because of the requirement for greater irradiance to achieve semisaturation, is less prominent with low-irradiance stimulation. This occurs even though the maximal amplitude of the R-cones (*V*_rmax_) is always greater (Figs. 5A, 5B, and 6C).

With the IR background, the roy adult spectral curves are greater in amplitude than with red or blue backgrounds (Fig. 6B). In the model parameters (Fig. 6C), this results mainly from lower semisaturation of R-cone signals (log *k*_r_) on IR backgrounds as compared with either blue or red backgrounds [ANOVA, *F*(2, 3847) = 268.8, *P* < 0.0000]. Lower semisaturation of G-cone signals (log *k*_g_) [ANOVA, *F*(2, 3847) = 21.7, *P* < 0.0000] also contributes to greater-amplitude spectra on IR backgrounds. As compared with IR backgrounds, B-cone signals are lost on blue backgrounds and somewhat desensitized on red backgrounds (*t*-test, *df* = 3078, *P* < 0.01).

In adults, as in larvae, the spectral shape extends to longer wavelengths with blue or IR backgrounds than with red backgrounds (Fig. 6B). As with larvae, one can examine whether R1 and R2 cones, differentially desensitized by the 627-nm red adapting beam, play a role, or whether the change in spectral shape reflects selective R-cone desensitization as compared with G-cones. The adult R-cone peak on red background is model fit at 566.0 ± 2.2 nm (Fig. 6C, adult), the R-cone peak on blue background is model fit at 574.9 ± 1.6 nm (Fig. 6C, adult), and the IR peak at 571 ± 2 nm is intermediate. The R-cone peaks on red and blue backgrounds differ significantly (*t*-test, *df* = 1748, *P* < 0.01). This suggests there are two opsins in separate adaptation pools, such as R1 and R2 cones, both contained within the red (535–610 nm) waveband. The longer wavelength R1 cone, containing longer wavelength LWS1 opsin (Chinen et al., 2003), is more desensitized by the 627-nm red background, than the R2 cone, containing LWS2 opsin. The net red-background effect in roy adults is to shift the apparent opsin peak toward the shorter-wavelength-peaking R2. In addition to R-cone desensitization by red backgrounds, as reflected in the increased R-cone semisaturation irradiances (log *k*_r_), an opsin shift from the longer wavelength R1 to the shorter-wavelength R2 also contributes. These spectral data support a mixture R1 and R2 cones with LWS1 or LWS2 opsins, respectively, in the adult eyecup.

The model-fit G-cone opsin peak is 483 ± 3 (*n* = 980) on the red background, 477 ± 6 (*n* = 770) on the blue background, and 482 ± 5 (*n* = 2100) on the IR background. These did not significantly differ [ANOVA, *F*(2, 3847) = 0.28, *P* = 0.758]. This may indicate only one of the four RH2 opsins is dominant in adult, roy, G-cones, perhaps RH2-3 (G3 cones). No 500 nm rhodopsin peak appeared on the IR background, as the stimulus protocol itself kept rods in a light-adapted state.

### Development of opsin peaks

The multiple opsin genes in zebrafish are expressed in a developmental and spatial sequence (summarized in Fig. 1). The results from *in situ* hybridization (Takechi & Kawamura, 2005) predict a migration of both R-cone and G-cone peaks from shorter to longer wavelengths over the course of cone development but predict no change in U-cone or B-cone spectral patterns. Here, we use ERG PIII spectral analysis to test these predictions.

#### Opsin peaks in the UV waveband

**Fig. 7.**
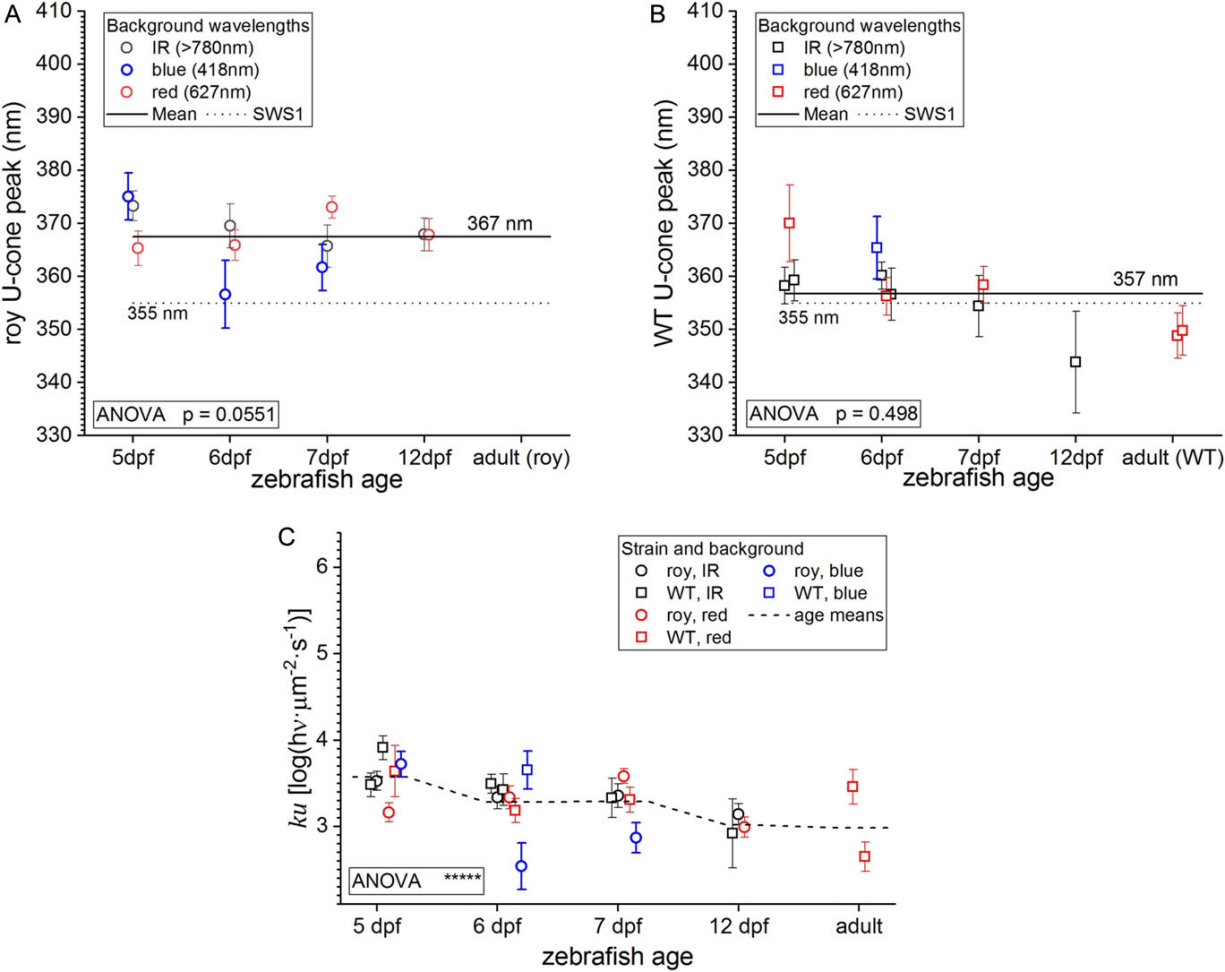
Within the UV waveband (330–389 nm), U-cone peaks are stable during development. (**A**, **B**) U-cone peaks (±s.e.) are calculated by fitting normalized, treatment-level datasets from many eyes at each age and background wavelength to eqn. (3). In this and succeeding figures, if no peak was found, no data point is plotted. Results are compared with the 355 nm SWS1 opsin peak in solution (dotted line) (Chinen et al., 2003) and to the all-age means (solid lines). Red points, 627 nm background; blue points, 418 nm background; black points, *λ* > 780 nm (IR) background. In (**A**) UV opsin peaks are fit for the roy strain. No roy adult UV opsin peaks could be fit on any background. Comparing the roy larval ages, no significant differences in opsin peaks occurred among the treatment groups [ANOVA, *F*(10, 14,759) = 1.80, *P* = 0.055, eyes = 140]. The mean of all fit values, including all larval ages and background illuminations, was 367.4 ± 1.6 nm (s.e., *n* = 11 fits to normalized, treatment-level datasets ranging from 700 to 1960 points each). In (**B**) UV opsin peaks are fit for WT. In WT adults, UV peaks were fit, but only with red, 627-nm backgrounds. From all larval stages through adulthood, no significant differences in opsin peaks were found [ANOVA, *F*(11, 10,760) = 0.94, *P* = 0.498]. The mean of all WT fit values (larvae and adults, three backgrounds) was 356.8 ± 2.0 nm (s.e., *n* = 12 fits to normalized treatment-level datasets ranging from 700 to 1960 points, solid line). The two adult points are separate datasets collected by MT and TS in different years. (**C**) UV-cone semisaturation irradiances *k*_u_, as measured in units of log(hν·μm^−2^·s^−1^), where hν is a symbol for quanta, decrease to adult levels during larval development, as the UV cones, a low semisaturation type in zebrafish retina, continue to gain sensitivity. Semisaturations differed [ANOVA, *F*(22, 25,659) = 4.1, *P* < 0.00001] with 13 of 15 Tukey post-hoc pairing different ages (0.05 > *P* < 0.0001), suggesting the illustrated trend is significant.

Based on molecular studies, there is a single UV opsin expressed both in larvae and in adults [Fig. 1, (Chinen et al., 2003; Takechi et al., 2008)]. U-cone signals are particularly large in larvae but weak in adults (Fig. 5B). For roy larvae, there were 11 large, normalized treatment-level larval datasets at different ages, and under different background conditions that yielded values for UV waveband opsin peaks (Fig. 7A). Recordings from 140 larval eyes produced 14,770 points total in these datasets, ranging from 700 to 1960 points in each one. No U-cone peak was fit in adult roy datasets (28 eyes). The mean of fits to roy U-cone peaks at all larval ages and background illuminations was 367.4 ± 1.6 nm (s.e., *n* = 11 treatment-level datasets). No significant difference was found among the 11 U-cone peak values [ANOVA, *F*(10, 14,759) = 1.80, *P* = 0.055], and there is no appearance of a developmental shift in the peak (Fig. 7A).

U-cone signals in adult WT zebrafish have been elsewhere reported (Bilotta et al., 2005; Hughes et al., 1998; Nelson & Singla, 2009), so we examined fish of WT coloration (Fig. 7B). There were 12 normalized, compiled WT datasets with fit values for U-cone peaks (10,772 points total, dataset minimum size, 61 points, maximum size, 3220 points, 67 eyes). Among these, U-cone peaks were fit in adults for two treatment-level datasets, acquired by different authors at different times (18 eyes total). These peaks were only discerned in the presence of red backgrounds, which reduced R-cone sensitivity. In WT eyes, from larval through adult stages, regardless of background wavelength, no significant difference was found among U-cone spectral peaks [ANOVA, *F*(11, 10,760) = 0.93, *P* = 0.498], arguing against a developmental progression. The mean fit of WT U-cone peaks was 356.8 ± 2.0 nm (s.e., *n* = 12 datasets). This is a significantly shorter peak wavelength than found for roy U-cones (367.4 ± 1.6 nm) (*t*-test, *t* = 4.03, *df* = 21, *P* < 0.001).

U-cone sensitivity, as measured by semisaturation (*k*_u_), differs across zebrafish development [ANOVA, *F*(22, 25,659) = 4.0, *P* < 0.00001, Fig. 7C]. Among significantly different Tukey post hocs, 12 of 14 were pairs from different age groups. Semisaturation in both WT and roy strains decreased slightly with age, reaching adult levels by day 12 (Fig. 7C). The value of log *k*_u_, in log(quanta·μm^−2^·s^−1^), decreases from 3.57 ± 0.10 at 5 dpf to 3.0 ± 0.06 at 12 dpf (Fig. 7C). Semisaturation is about the same, regardless of background wavelengths used, or the larval strain. Semisaturation irradiance is characteristic of cone type. U-cones are a low semisaturation type throughout development, particularly as compared with R-cones (see below).

#### Blue-waveband opsin peaks

**Fig. 8.**
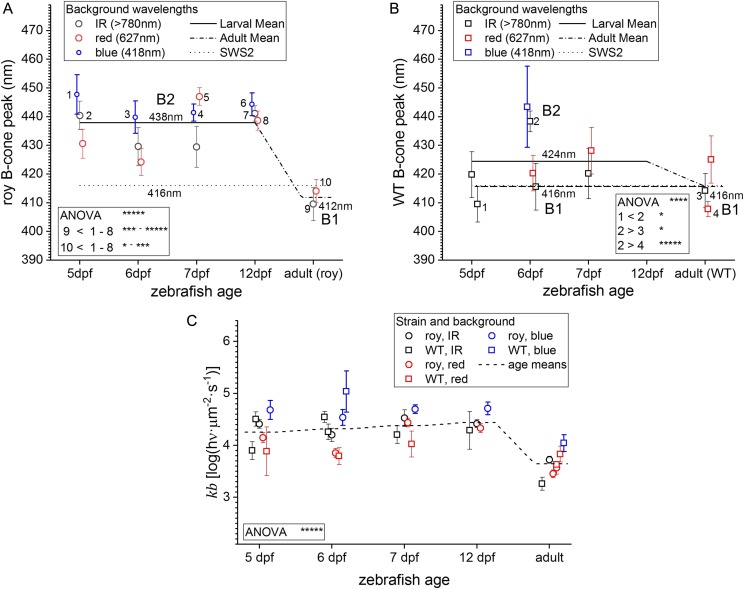
B-cone peaks within the blue-waveband (390–449 nm) shift to shorter wavelengths with development. (**A**, **B**) B-cone peaks (±s.e.) are calculated by fitting normalized, treatment-level datasets from many eyes at each age and background wavelength to eqn. (3). Results are compared with the 416 nm SWS2 opsin peak in solution (dotted line) (Chinen et al., 2003) and with the B-cone means for 5–12 dpf larvae (solid lines) and for adults (dash-dot lines). Red points, 627 nm background; blue points, 418 nm background, black points, *λ* > 780 nm. (**A**) B-cone peaks are fit for the roy strain. Comparing all ages, the peaks differed significantly [ANOVA, *F*(13, 19,726) = 6.61, *P* < 0.00001, eyes = 169]. Sixteen larval–adult dataset pairs differed (Tukey, 0.05 > *P* < 0.00001, legend). No larval–larval or adult–adult dataset pairs differed (*P* > 0.05). The mean larval B-cone peak value (B2) was 437.9 ± 2.2 nm (solid line, 12 treatment-level datasets), the adult (B1), 411.8 ± 2.3 nm (s.e., 2 treatment-level datasets). Datasets ranged from 700 to 2100 points each. (**B**) B-cone peaks are fit for WT. Comparing all ages, opsin peaks differed significantly [ANOVA, *F*(10, 11,858) = 3.59, *P* < 0.00001, eyes = 77]. Two larval–adult dataset pairs differed (Tukey, *P* < 0.01), each comparing the 6 dpf IR background value with an adult value. One larval–larval pair differed (B1, B2, see legend). The larval mean fit value was 424.4 ± 4.1 nm (s.e., B1, B2, solid line, mean of eight treatment-level datasets), the adult, B1-peak, 415.7 ± 5.0 nm (s.e., *n* = 3 treatment-level datasets). Adult normalized treatment-level datasets contained from 139–3220 points. (**C**) B-cones gain sensitivity in adulthood. B-cone semisaturation irradiances *k*_b_ [in log(hν·μm^−2^·s^−1^)] increase slightly during the larval period but decrease sharply after the transition to adulthood, making a transition from high semisaturation larval B1/B2 cones, to low semisaturation adult B1 cones. Semisaturations differed [ANOVA, *F*(28, 34,472) = 13.1, *P* < 0.00001] with 82 of 102 Tukey post hoc pairing a larval with an adult dataset, an evidence of developmental shift. The trend for increased *k*_b_ values with blue adaptation also appears significant, with 11 of 102 Tukey post hoc pairing red- and blue-adapted larval datasets (0.05 > *P* < 0.0001).

Fourteen normalized, treatment-level datasets (700–2100 points each) yielded B-cone peaks for the roy strain (Fig. 8A). These included 169 eyes yielding 19,740 amplitude-wavelength-irradiance points. Due to desensitization, no adult B-cone peak was fit in the blue-adapted roy dataset (six eyes). The variation in B-cone peaks during development was significant [ANOVA, *F*(13, 19,726) = 6.61, *P* < 0.00001]. Tukey post hoc tests reveal 16 peak pairs that differed (0.05 > *P* < 0.00001). All paired a shorter-wavelength adult B-cone with a longer-wavelength larval B-cone. This suggests that there are two B-cone populations, the longer-wavelength larval B2 cone, and the shorter-wavelength adult B1-cone. The mean roy B2 larval peak, including all background treatments at 5–12 dpf, is 437.9 ± 2.2 nm (s.e., *n* = 12 datasets). The mean adult B1 peak (IR and red backgrounds) is 411.8 ± 2.3 nm (s.e., *n* = 2 datasets).

There is significant dispersion of blue-waveband B-cone peaks in WT eyes but less of a developmental progression (Fig. 8B). There are 11 treatment-level datasets for different WT ages and background illumination (139–3220 points, 77 eyes) that yielded blue waveband peaks. Differences in peaks were significant [ANOVA, *F*(10, 11,847) = 3.59, *P* < 0.0001). Tukey post-hoc tests reveal three opsin peak pairs that differed (0.05 > *P* < 0.0001). In two, an adult B1 cone peak lays at a shorter wavelength than a larval B2 cone peak. In the third, two larval B-cone peaks differed. The mean WT larval peak, 5–12 dpf, including all background treatments, is 424.4 ± 4.1 nm (s.e., *n* = 8 datasets). The mean adult peak (IR and red backgrounds) is 415.7 ± 5.0 nm (s.e., *n* = 3 datasets). The results suggest that at least two cones, B1 and B2, inhabit the blue waveband in WT larvae, but that only B1 cones are seen in either WT or roy adults.

B-cone sensitivity, as measured by semisaturation (*k*_b_), changes during zebrafish development [ANOVA, *F*(28, 34,472) = 13.1, *P* < 0.00001, Fig. 8C]. Of 102 significantly different Tukey post hocs, 82 paired high semisaturation larval B1 or B2 cones with the low semisaturation adult B1 cones. The log *k*_b_ value for larvae (all treatments and backgrounds) was 4.34 ± 0.07, and that for adults was 3.64 ± 0.10. A shift to lower B-cone semisaturation occurs after the larval period (Fig. 8C). Among significantly different Tukey post hocs, 11 paired semisaturations on red and blue backgrounds. The mean larval log *k*_b_ value on red backgrounds was 4.07 ± 0.09, and that for blue backgrounds was 4.73 ± 0.08. This is consistent with selective adaptation of the B1 cone by the 418 nm background.

Taken together, results suggest that both a shorter-wavelength B1 cone and a longer-wavelength B2 cone operate in the blue-opsin waveband (390–449 nm) and that both B1 and B2 cones are expressed in larvae, but B1 cones predominate in adults. Further, the larval B-cones, while producing signals of significant amplitude (Fig. 5B) require more light than adult B-cones. It is interesting that blue (418 nm) backgrounds tend to shift the larval opsin peaks to the longer wavelengths. This latter effect is suggestive of separate adaptation pools for the two larval blue opsins, that is, B1 and B2 cones.

#### Green-waveband opsin peaks

**Fig. 9.**
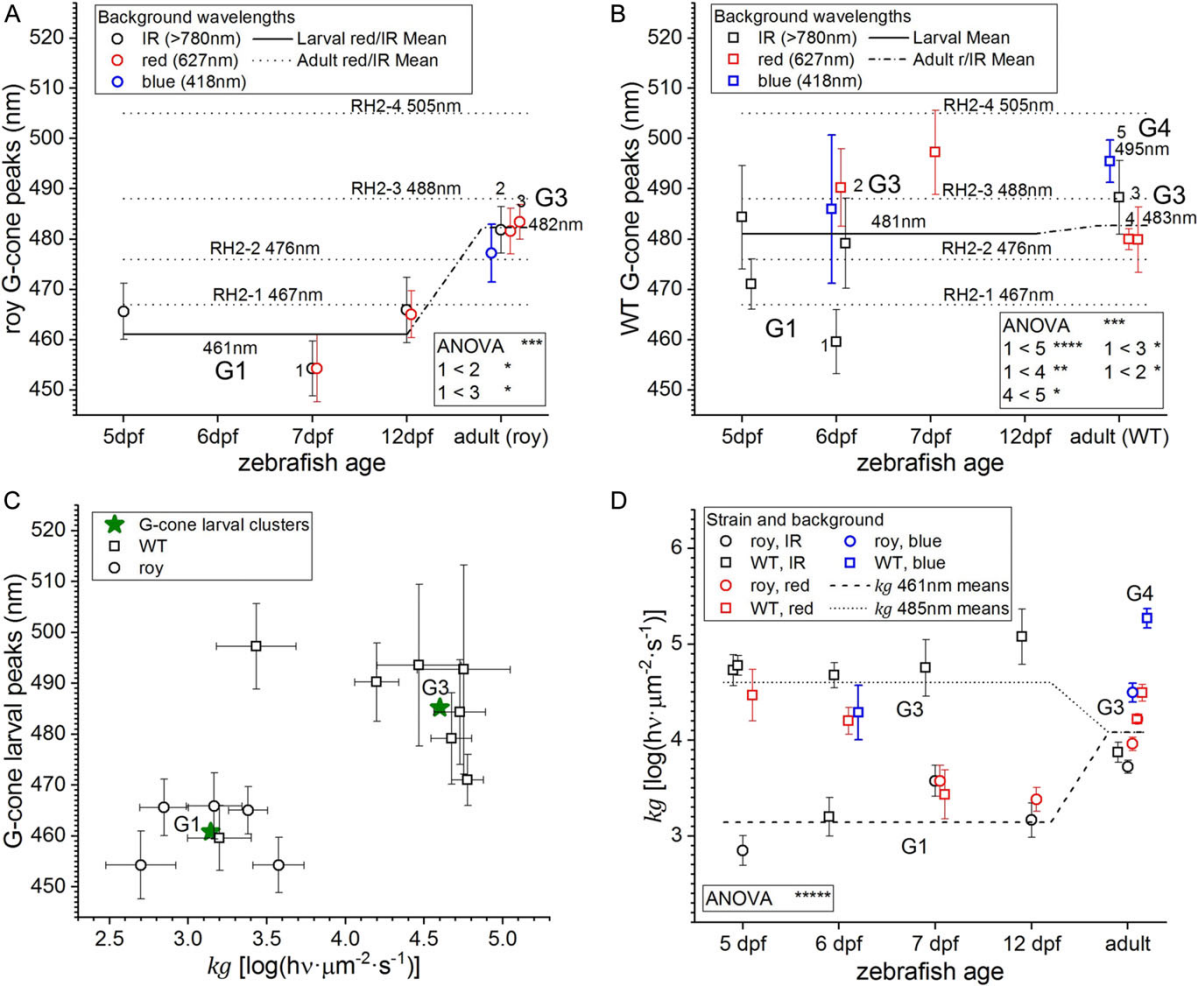
Development of G-cone peaks in the green waveband (450–529 nm). (**A**, **B**) G-cone peaks (±s.e.) are calculated by fitting normalized, treatment-level datasets from many eyes at different ages and background wavelengths to eqn. (3). Results are compared with the RH2-1 (467 nm), RH2-2 (476 nm), RH2-3 (488 nm), and RH2-4 (505 nm) opsin peaks in solution (dotted lines) (Chinen et al., 2003) and with the means on all backgrounds for 5–12 dpf larvae (solid lines) and adults (dash dot lines). (**A**) G-cone peaks are fit for the roy strain. Comparing all ages, significantly different G-cone peaks occurred [ANOVA, *F*(8, 13,081) = 3.84, *P* < 0.001, eyes = 113]. The two significant Tukey post hoc tests (*P* < 0.05, see legend) found a larval G1-cone peak at shorter wavelength than an adult G3 cone peak. The larval mean G1 peak was 461.0 ± 2.8 nm, the adult G3 peak, 482.3 ± 0.6 nm (s.e., *n* = 5 larval and 3 adult treatment-level datasets ranging from 980–2100 points, red and IR backgrounds). (**B**) G-cone peaks are fit for WT. Comparing all ages, significant differences in opsin peaks occurred [ANOVA, *F*(10, 12,747) = 3.8, *P* < 0.0001, eyes = 80]. Three significant Tukey post hoc tests found a larval G1 cone peak at shorter wavelengths than an adult G3 or G4 (0.05 > *P* < 0.00001, see legend). Adult G3 and G4 peaks differed (red *vs.* blue backgrounds, see text, *P* < 0.05). Larval G1 and G3 cone peaks differed (red *vs.* IR backgrounds, Tukey, *P* < 0.05, see legend). The larval mean fit value was 481.1 ± 4.7 nm (s.e., *n* = 7 treatment-level datasets, ranging from 139 to 1858 data points, red, blue, and IR backgrounds). The adult G4 peak fit on blue background was 495.5 ± 4.2 nm (s.e., one treatment-level dataset, 1469 points). The adult G3 peak on the red and IR backgrounds was 482.7 ± 2.7 nm (s.e., three treatment-level datasets, 1258 to 3220 points). (**C**) Larval G-cone wavelength peaks and semisaturation values form G1 and G3 cone clusters. G1 cones are shorter wavelength and lower semisaturation than G3 cones. G1 occurs mainly in roy larvae; G3, in WT larvae. (**D**) Semisaturation (*k*_g_) for both G1 and G3 cones are stable during larval development. Lines are means of sensitivities on red, blue, and IR backgrounds for G1 and G3 cones. Semisaturations differed [ANOVA, *F*(22, 26,708) = 25.1, *P* < 0.00001] with 38 of 187 Tukey post hoc pairing larvae (0.05 > *P* < 0.0001), suggesting G1 and G3 larval cone types differ in sensitivity.

With four RH2 opsins (Chinen et al., 2003), rods (Fadool, 2003), and a relatively low signal level in larvae (Fig. 5B), G-cone peaks are an interpretive challenge for the spectral model. Based on *in situ* hybridization, a shift from shorter- to longer-wavelength opsins during development is anticipated (Takechi & Kawamura, 2005). In roy datasets, all computed G-cone peaks are within the range of RH2 opsin peaks in solution (Chinen et al., 2003), and a progression to longer wavelengths in adults is in fact observed (Fig. 9A). Nine normalized, treatment-level datasets (770–2100 points each) yielded green opsin peaks (Fig. 9A). These included 113 eyes with a total of 13,090 amplitude-wavelength-irradiance points. The developmental progression in G-cone peaks among treatment-level datasets was significant [ANOVA, *F*(8, 13,081) = 3.84, *P* < 0.001]. Tukey post hoc tests reveal two G-cone peak pairs that differed (1 < 2, 1 < 3, *P* < 0.05), each contained a longer-wavelength adult G3 value and a shorter-wavelength larval G1 value. The mean G1 larval peak (5–12 dpf, IR and red backgrounds) is 461.0 ± 2.8 nm (s.e., *n* = 5 treatment-level datasets). In four, larval, blue-background treatment-level datasets, no G-cone peaks were found. The mean G3 adult peak (IR and red backgrounds) is 482.3 ± 0.6 nm (s.e., *n* = 3 datasets).

G-cone peaks computed from the WT datasets fell within the range of RH2 opsins (Chinen et al., 2003), but with more scatter, and little developmental shift evident (Fig. 9B). Eleven treatment-level datasets (139–3220 points each) yielded green opsin peaks. These included 80 eyes with a total of 12,758 amplitude-wavelength-irradiance points. The variation in WT G-cone peaks was significant [ANOVA, *F*(10, 12,747) = 3.14, *P* < 0.001]. Tukey post-hoc tests reveal four opsin peak pairs that differed, and an additional pair was revealed by a *t*-test (0.05 > *P* < 0.0001). An adult G3 spectral peak was longer wavelength than a larval G1 spectral peak in two cases (1 < 3, 1 < 4, Fig. 9B). An adult G4 spectral peak was longer wavelength than a larval G1 peak in one case (1 < 5). A larval G1 peak was shorter wavelength than a larval G3 peak in one case (1 < 2). In a fifth case (4 < 5), an adult G4 peak, found in a blue-adapted dataset, was longer wavelength than an adult G3 peak found in a red-adapted dataset. As these latter adult datasets were obtained on the same set of eyes, a *t*-test (*t* = 2.06, *df* = 2725, *P* < 0.05) was used to test significance. The longer-wavelength G4 peak was not seen in blue-adapted roy adults (Fig. 9A). The mean WT G-cone larval peak (5–12 dpf, all background treatments) is 481.1 ± 4.7 nm (s.e., *n* = 7 treatment-level datasets) and contains a mixture of G1 and G3 cone peaks. The mean adult G3 peak on red or IR background is 482.7 ± 2.8 nm (s.e., *n* = 3 treatment-level datasets). The adult G4 peak is 495.5 ± 4.2 nm (*n* = 1 treatment-level dataset of 1469 points).

A scatter plot of larval G-cone wavelength peaks and green-cone semisaturation (*k*_g_) reveals two clusters: a shorter-wavelength G1 cone with low-irradiance semisaturation and a longer-wavelength G3 cone with high-irradiance semisaturation (Fig. 9C). The G3 cone appears mainly in the treatment-level datasets from WT larvae, while the G1 cone appears in both roy and WT larvae. Taking means from values obtained on red or IR backgrounds, the G1 wavelength peak is 460.8 ± 2.1 nm (s.e., *n* = 6 datasets) and the G3 peak is 485.2 ± 3.1 nm (s.e., *n* = 6 datasets).

The development of semisaturation levels (*k*_g_, Fig. 9D) suggests a bifurcated plot. Semisaturation values differ [ANOVA, *F*(22, 26,708) = 25.1, *P* < 0.0001]. The Tukey post hoc test shows 187 pairs with significant differences, with 38 of them between larvae, consistent with both G1 and G3 cone sensitivity types. In adults, a single dominant semisaturation type appears. It is a longer wavelength G3 type but with a semisaturation (*k*_g_) intermediate between larval G1 and G3 types. Excluding the G4 value on blue backgrounds, only 1 of the 187 significantly different Tukey pairs was between two adult values. The mean log of semisaturation for the larval G1 cone (G1, Fig. 9D) is 3.1 ± 3.1 0.1 log(quanta·μm^−2^·s^−1^), that of the longer-wavelength larval G3 cone (G3, Fig. 9D) is 4.6 ± 0.1 log(quanta·μm^−2^·s^−1^), and that of the adult G3 cone is 4.1 ± 0.1 log(quanta·μm^−2^·s^−1^). The semisaturation of the WT adult G4 cone on blue background is 5.3 ± 0.1 log(quanta·μm^−2^·s^−1^).

#### Red-waveband opsin peaks

**Fig. 10.**
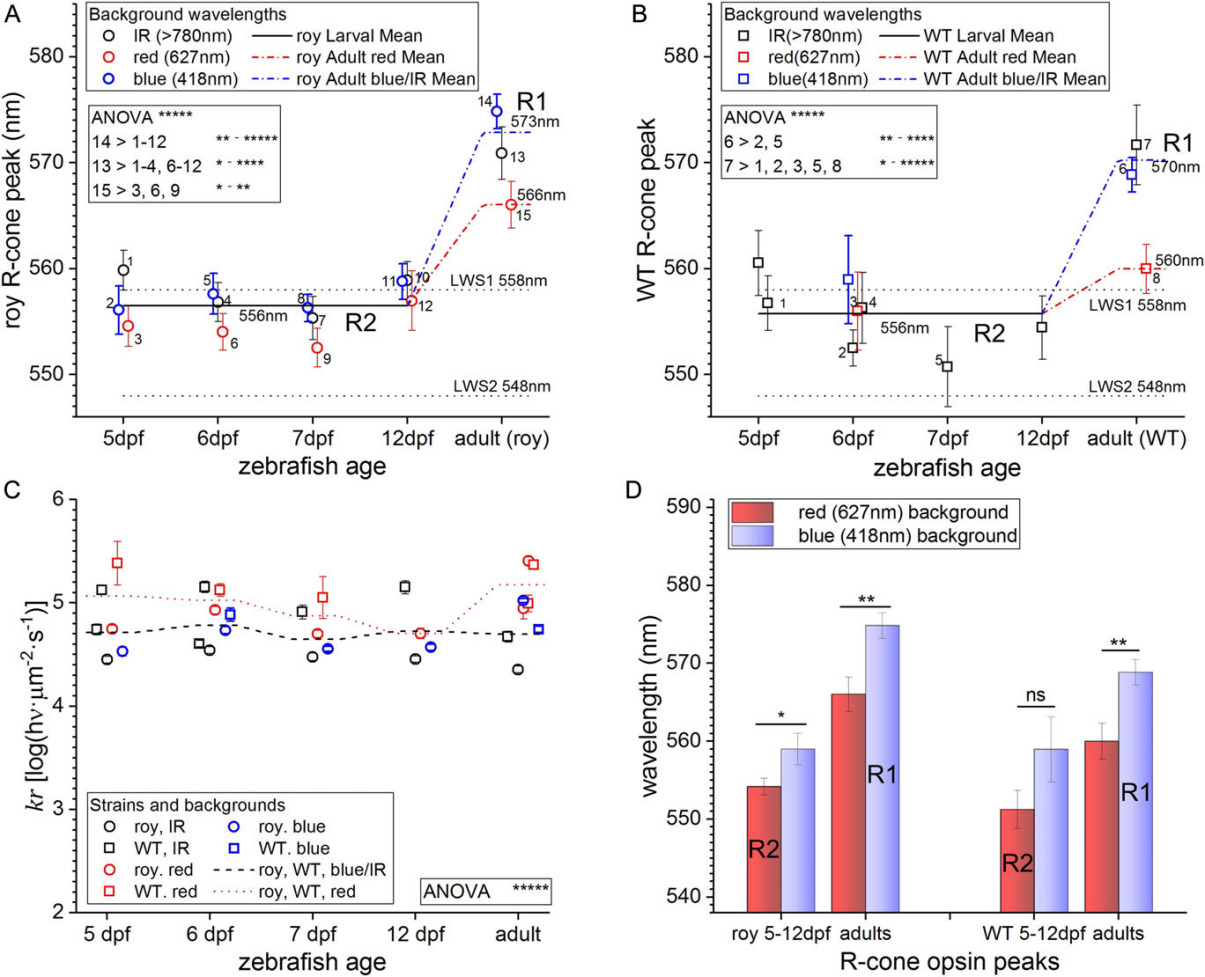
Developmental shift from R2 to R1 cones in the red-waveband (530–630 nm). (**A**, **B**) R-cone wavelength peaks (±s.e.) are calculated by fitting treatment-level datasets from many eyes at each age and background wavelength to eqn. (3). Results are compared with the 558-nm LWS1 and 548-nm LWS2 opsin peaks in solution (dotted lines) (Chinen et al., 2003), with the 5–12 dpf larval (R2-cone) means (solid lines), and with the adult (R1) cone means on red (627 nm) *vs.* combined blue (418 nm) and IR (*λ* > 780 nm) backgrounds (red and blue dash-dot lines respectively). (**A**) R-cone peaks for the roy strain differed with age and background [ANOVA, *F*(14, 19,375) = 8.42, *P* < 0.00001, eyes = 113]. All 26 significant Tukey post hocs (0.05 > *P* < 0.00001) paired a shorter-wavelength larval R2 peak with a longer-wavelength adult R1 peak. The larval mean fit value was 556.5 ± 0.6 nm [s.e., *n* = 12 normalized treatment-level datasets ranging from 700 to 1960 points each, including red, blue and IR backgrounds]. The mean adult R2-cone peak on IR and blue backgrounds was 572.9 ± 2.0 nm (s.e., *n* = 2 treatment-level datasets, 770 and 2100 points) and on red background, 566.0 ± 2.5 nm (s.e., 1 treatment-level dataset, 980 fit points). (**B**) WT R-cone wavelength peaks depend on developmental stage and background illumination [ANOVA, *F*(10, 10,210) = 6.1, *P* < 0.00001, eyes = 79]. All seven significant post hoc pairs found a larval R2 peak at shorter wavelengths than an adult R1 (Tukey, 0.05 > *P* < 0.00001).The larval R2 mean fit value was 555.8 ± 1.1 nm (s.e., *n* = 8 larval treatment-level datasets, ranging from 210 to 1858 data points, including red, blue, and IR backgrounds). The mean adult R2 peak (blue and IR backgrounds) was 570.3 ± 1.4 nm (s.e., two treatment-level datasets, 1469 and 1400 points) and on a red background, 560.0 ± 2.3 nm (s.e., one treatment-level dataset, 1258 points). (**C**) R-cones are high semisaturation types at all ages in both WT and roy. Semisaturations differed (ANOVA, see text), but without developmental trend. Lines show minimal age variations on red backgrounds (red dots) or blue and IR combined backgrounds (black dashes). Semisaturation on red backgrounds is greater (see text). (**D**) R-cone peaks shift to shorter wavelengths on red backgrounds as compared with blue. Larval R2 cone peaks are fits to composite 5–12-dpf treatment-level datasets on 627 nm background (roy, 5040 points; WT, 939 points) or 418 nm background (roy, 4060 points; WT, 210 points). In roy larvae and adults, and WT adults, the wavelength shift is significant (*t*-tests, see text). The red-background, short-wavelength shift suggests mixtures of R1 and R2 cones in both larvae and adults, with R2 dominant in larvae and R1 in adults.

R-cone saturation amplitudes are larger than those of any other cone type in both larvae and adults (Fig. 5B). In the red waveband, R-cone signals suffer little interference from other cone signals. Both these factors increase fit reliability and reduce fit errors for R-cone peaks. There are two red opsin genes *LWS1* and *LWS2* (Chinen et al., 2003). Based on message expression during development, the shorter-wavelength LWS2 opsin is expected to be dominant in larvae, followed by increasing expression of the longer-wavelength LWS1 opsin in juveniles and adults (Takechi & Kawamura, 2005). In larval physiology, a shorter-wavelength R2 cone dominates both roy and WT larvae (556 nm, Fig. 10A and 10B). In adult physiology, a longer-wavelength R1 cone is predominant (570–575 nm).

For the roy coloration strain, 15 normalized, treatment-level datasets (700–2100 points each) yielded R-cone peaks (Fig. 10A). These included 176 eyes with a total of 19,390 amplitude-wavelength-irradiance points. The developmental progression among R-cone peaks was significant [ANOVA, *F*(14, 19,375) = 8.42, *P* < 0.00001]. Twenty-six Tukey post hoc tests paired a longer-peak-wavelength adult R1 cone with a shorter-peak-wavelength larval R2 cone (0.05 > *P* < 0.00001; see Fig. 10A legend). No significantly different pairs were found between the 12 larval treatment-level datasets. The mean roy R2 larval peak (5–12 dpf, all background treatments) is 556.5 ± 0.6 nm (s.e., *n* = 12 treatment-level datasets). The mean roy adult R1-cone peak on blue or IR background is 572.9 ± 2.0 nm (s.e., *n* = 2 treatment-level datasets, 24 eyes). The mean adult R1 cone peak on red background is 566.0 ± 2.2 nm (s.e., *n* = 1 treatment-level dataset, six eyes).

The development of red-waveband, R-cone peaks for WT is similar to roy. Eleven normalized, treatment-level datasets (210–1858 points) yielded R1/R2 cone peaks (Fig. 10B). These included 79 eyes with a total of 10,221 amplitude-wavelength-irradiance points. Developmental change among R-cone peaks was significant [ANOVA, *F*(10, 10,210) = 6.13, *P* < 0.00001]. Eight significant Tukey post hoc paired shorter-wavelength larval R2 cone peaks with longer-wavelength adult R1 cone peaks (Fig. 10B, legend). One paired two adult R cone peaks on different backgrounds (red *vs.* IR, *P* < 0.05). As with roy, a definite long-wavelength shift in R-cone peak occurs between larvae and adults, and in addition, between adult R1 cone peaks on chromatic backgrounds. The mean WT larval R2 cone peak (5–12 dpf, all background treatments) is 555.8 ± 1.1 nm (s.e., *n* = 8 datasets). The mean adult R1 peak on blue or IR backgrounds is 570.3 ± 1.4 nm (s.e., *n* = 2 datasets, 22 eyes). The mean adult R1 peak on red background is 560.0 ± 2.3 nm (s.e., *n* = 1 dataset, 11 eyes).

There are significant differences in semisaturation levels for R-cones [ANOVA, *F*(29, 35,241) = 19.2, *P* < 0.00001)]; nonetheless, there is little evidence of developmental progression (Fig. 10C). The mean value at all ages on blue and IR backgrounds is 4.7 ± 0.06 log(quanta·μm^−2^·s^−1^) (s.e., *n* = 18 datasets), making both R1 and R2 cones high semisaturation types throughout development. On red background, the mean semisaturation increases to 5.0 ± 0.08 log(quanta·µm^−2^·s^−1^) (s.e., *n* = 11 datasets), a significantly red-desensitized value (*t*-test, *df* = 27 datasets, *P* < 0.01).

Red (627 nm) background illumination shifted R-cone peaks to shorter wavelengths with respect to blue (418 nm) background illumination in both larvae and adults. To better illustrate the shifts in R-cone peaks that occur in larvae, spectral datasets from 5–12 dpf larvae are combined (Fig. 10D). Three of the four red background, blue background comparisons differed (roy larvae, *t*-test, *t* = 2.2, *df* = 9098, *P* < 0.05; roy adults, *t*-test, *t* = 3.05, *df* = 1748, *P* < 0.01; WT larvae *t*-test, *t* = 1.4, *df* = 1147, *P* = 0.16; WT adults, *t*-test, *t* = 3.20, *df* = 2725, *P* < 0.01). For WT larvae, although the short-wavelength shift with red backgrounds was not significant, the trend was the same. The all-age larval datasets suggest that, both for adults and, likely, for larvae, two independent R1 and R2 cone adaptation pools are physiologically active. Nonetheless, the larval R-cone mixture is predominantly the shorter-wavelength R2 cone (containing LWS2 opsin) seen peaking between 551 and 554 nm on red backgrounds. The adult R-cone mixture trends toward the longer-wavelength R1 cone (containing LWS1 opsin), peaking between 570 and 575 nm on IR or blue backgrounds, which do not selectively desensitize R1 cones.

### Cone types in larvae and adults

**Fig. 11.**
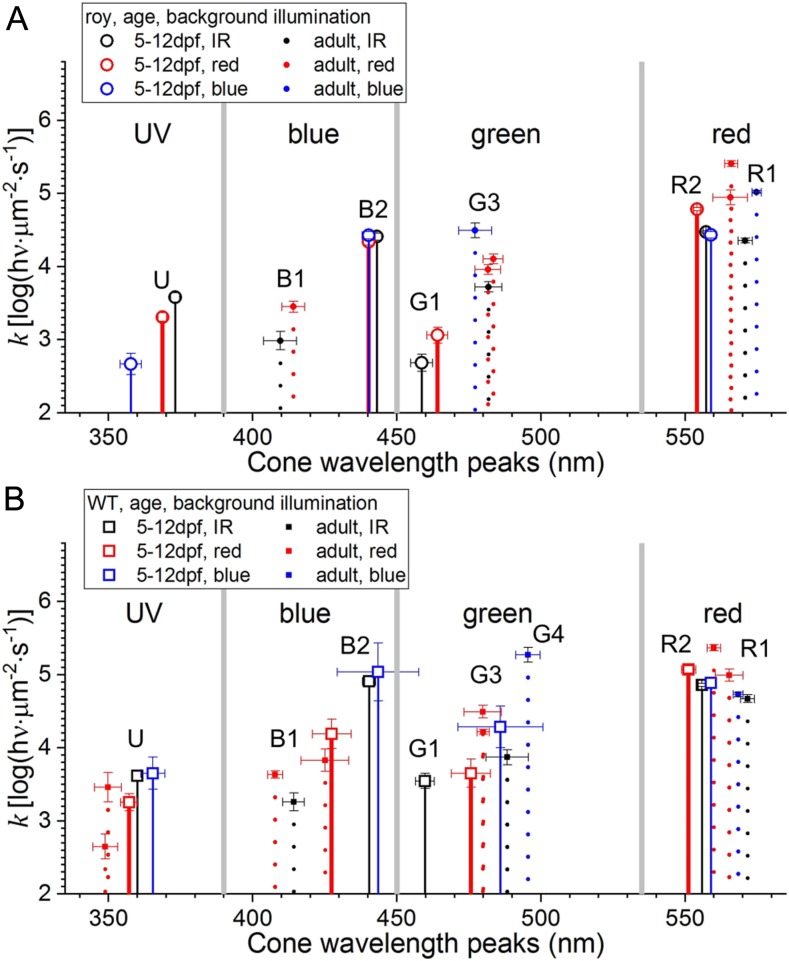
Eight larval and adult cone types (U, B1, B2, G1, G3, G4, R2, R1) are identified by wavelength peak and semisaturation irradiance. These cone-specific values are fit by the spectral model [eqn. (3)] to normalized, treatment-level datasets. For larvae (open symbols, solid drop lines), these datasets combine 5–12 dpf ages, yielding an overall larval profile. Adult cone properties (solid symbols and dotted drop lines) are a replotting of the treatment-level datasets previously shown. The symbol and drop line colors indicate wavelengths of selective chromatic adaptation (red, 627 nm; blue, 418 nm; black, *λ* > 780 nm). In the model, opsin peaks are fit within wavebands (UV, blue, green, and red, bounded by vertical gray bars). (**A**) In roy, seven cone types are detected overall. In roy larvae, there are two low semisaturation cone types (U and G1) and three high semisaturation types (B2, G3, and R2). Four types appear in roy adults: one is low semisaturation (B1) and three are high semisaturation (G3, R1, and R2). Three prominent cone-type shifts occur between larvae and adults: B2 to B1, G1 to G3, and predominantly R2 to predominantly R1. (**B**) In WT, eight cone signals are detected, including the seven seen in roy, and adding G4. At both larval and adult stages, WT shows more cone peaks than roy. There is an adult WT (but not roy) U-cone. There is a G3 cone in WT, but not roy, larvae. There is a G4 cone in WT but not in roy adults. The number of points fit for the 5–12 dpf roy larvae on each background wavelength are IR, 6440; red, 5040; blue, 4060. For WT 5–12 dpf larvae, the values are IR, 5145; red, 939; blue, 210. The number of points fit for the adult cone types is previously listed.

Wavelength peaks and semisaturation irradiances characterize cone types (Fig. 9C). As seen in Figs. 7–10, there is only modest variation in cone wavelength peak or semisaturation during the 5–12 dpf larval period. This suggests that larval datasets can be combined into a single, all-age, treatment-level dataset for larvae. This provides a larval–adult overview (Fig. 11A and 11B). Here, the red, green, blue, and UV wavebands are separated by vertical gray bars (Fig. 11A and 11B). Larval cones (solid drop lines) and adult cones (dotted drop lines) are characterized by cone wavelength peaks and semisaturation values (*k*). In adults and larvae, taken together, there are eight cone peaks resolved, U (350–370 nm), B1 (410–415 nm), B2 (440 nm), G1 (460 nm), G3 (480 nm), G4 (490 nm), R2 (556 nm), and R1 (570–575 nm). G and R numbering is set to correspond with RH2 and LWS opsin numbering (Chinen et al., 2003).

The roy strain overall shows seven of eight cone types, five resolved in larvae and four in adults (Fig. 11A). WT express all eight, seven in larvae and six in adults (Fig. 11B). In the roy larval UV waveband, there is a clustering of values, depending on background illumination, between 360 and 370 nm. These are low semisaturation irradiance larval U-cones [2.7 −3.6 log(quanta·μm^−2^·s^−1^)]. In the roy blue-waveband, a larval B-cone with high semisaturation irradiance [4.4 log(quanta·μm^−2^·s^−1^)] is seen peaking at about 440 nm. Chromatic backgrounds do not reveal another peak, indicating a single dominant B2-cone type. The adult roy B1 cone contrasts with larval B2, being a low semisaturation type [3.5 log(quanta·μm^−2^·s^−1^)] peaking at 410–415 nm. In the roy larval green waveband, a low semisaturation G1 cone [2.7--3.1 log(quanta·μm^−2^·s^−1^)] is found peaking at about 460 nm, regardless of background wavelength. This contrasts with the roy adult G3 cone, which is a high semisaturation type [∼4 log(quanta·μm^−2^·s^−1^)] peaking at ∼480 nm, regardless of chromatic background. In the red waveband, for both larvae and adults, high semisaturation R1 and R2 cones prevail [4.4–5.4 log(quanta·μm^−2^·s^−1^)]. In roy and WT larvae, R2 peaks are tightly clustered between 550 and 560 nm, with less than 5 nm short-wavelength shift on red backgrounds. For roy larvae, this shift was significant (Fig. 10D). In roy and WT adults, the R1 peak (570–575 nm) can be shifted significantly toward the R2 peak with red chromatic adaptation (Figs. 10D, 11A, and 11B), indicating the presence of both R1 and R2 cones.

WT show more cone spectral peaks than roy at each developmental stage. Unlike roy adults, WT adults show low semisaturation U-cones and high semisaturation G4 cones (495 nm). In the WT blue-waveband, a high semisaturation larval B-cone is seen peaking at ∼440 nm, but, in addition, with red adaptation, a shorter-wavelength type emerges at 427 nm, perhaps a mixture of B1 and B2. The red-adapted B2 peak differs from the blue-adapted B2 peak (*t*-test, *t* = 2.1, *df* = 6082, *P* < 0.05). In single-age larval datasets, both 410 nm B1 and 438 nm B2 peaks are seen, and the peaks differed significantly (Fig. 8B). This suggests B1 and B2 cones both exist in WT larvae. Like roy, WT larval physiology shows a low semisaturation 460-nm-peaking G1 cone. Red chromatic adaptation reveals at least one other G-cone type, a ∼480-nm-peaking G3. It is like adult, with high semisaturation. The larval G1 and G3 peaks differ (*t*-test, *t* = 2.0, *df* = 6082, *P* < 0.05). In the red-waveband roy and WT larval cones, both shorter wavelength 550–560 nm R2 types are seen, with high semisaturation. The red-waveband roy and WT adult cones are mainly 570–575 nm R1 types, also with high semisaturation (Figs. 11A and 11B). Red adaptation significantly shifts R1 peaks toward shorter wavelengths, revealing the influence of adult R2 cones also.

### Model fits to PIII ERG datasets are comparable to MSP and patch recording in adult cones

For adult zebrafish, modeling results can be compared with MSP and patch spectral literature (Table 2). Previous measurements of opsin peak wavelengths were generally reported as means for morphological cone types. The presence of different opsins, such as LWS1 and LWS2, within a single cone type (Takechi & Kawamura, 2005; Tsujimura et al., 2010) was not evaluated. In Table 2, we compare the adult mean values obtained by the spectral model on neutral (IR) backgrounds as a measure comparable to MSP and patch recording. For adult R-cones, the mean literature peak from single-cell methods is 565.1 ± 1.8 nm (s.e., *n* = 8, Table 2). This does not differ from adult R-cone peaks, as inferred from spectral model fits to normalized, treatment-level cone-PIII spectral datasets on IR backgrounds (roy: 570.9 ± 2.5 nm, *t*-test, *t* = 0.14, *df* = 2106, *P* = 0.89; WT: 571.7 ± 3.7 nm, *t*-test, *t* = 0.13, *df* = 1406, *P* = 0.89; Table 2). The combined adult R-cone peaks obtained from MSP, patch recording, and the present computationally inferred values, however, lie at longer wavelengths than the LWS1 opsin solution extract value of 558 nm (Chinen et al., 2003) (one sample *t*-test, *t* = 5.18, *df* = 9, *P* < 0.01, Table 2). We conclude that values in solution and *in situ* differ.

**Table 2.** Comparison of present and literature values for adult zebrafish cone opsin absorbance peaks

Adult cone opsin peaks (nm)		Cone type			
Author	Method	UV	Blue	Green	Red
Levine et al. (1979)	MSP	nd	414	484	564
Nawrocki et al. (1985)	MSP	nd	417	480	558
Robinson et al. (1993)	MSP	360	415	480	570
Cameron et al. (2002)	MSP	nd	407	473	564
Allison et al. (2004)	MSP	361	411	482	565
Endeman et al. (2013)	Patch	365	416	483	574
Tarboush et al. (2012)	MSP	358	428	480	565
Enright et al. (2015)	MSP	nd	nd	nd	561
Literature [means ± s.e. (*n*)]	MSP patch	361 ± 1.5 (4)	415 ± 2.5 (7)	480 ± 1.4 (7)	565 ± 0.8 (8)
Chinen et al. (2003)	Cos-1 express	355	416	467/476	548/558
				488/505	
Spectral model [means ± s.e. (*n*)]	Cone PIII *roy orbison*	Not found	410 ± 5.8 (2100)^b^	482 ± 4.6 (2100)^b^	571 ± 2.5 (2100)^b^
	Cone PIII WT	349 ± 4.3 (3220)^a^	414 ± 6.0 (1400)^b^	488 ± 7.3 (1258)^b^	572 ± 3.7 (1400)^b^

*Note:* Microspectrophotometry (MSP), extracts of transfected Cos-1 cells (Cos-1 express), cone patch recordings (patch), spectral model analysis of cone PIII opsin peaks (Spectral model) in *roy orbison* and WT strains. Cone PIII is the ERG signal remaining after treating retinas with aspartate perfusion medium. (*n*), the number of data points fit by the spectral model or averaged in literature means.

a Red (627 nm) background.

b IR (*λ* > 780 nm) background.

For adult zebrafish G-cones, opsin peaks previously measured by MSP or patch recording averaged 480.3 ± 1.4 nm (s.e., *n* = 7, Table 2), similar to model fit values (roy: 481.8 ± 4.6, *t*-test, *t* = 0.02, *df* = 2105, *P* = 0.98; WT: 488.3 ± 7.3, *t*-test, *t* = 0.08, *df* = 1405. *P* = 0.94, IR background). All measures are within the ranges of RH2-1 to RH2-4 opsin peak values as found in solution (Chinen et al., 2003). Similarly, for adult blue cones, MSP and patch opsin peaks averaged 415.4 ± 2.5 nm (s.e., *n* = 7), not different from the model fits to adult cone-PIII datasets (roy: 409.5 ± 5.8 nm, *t*-test, *t* = 0.09, *df* = 2105, *P* = 0.95; WT: 414.2 ± 6.0, *t*-test, *t* = 0.01, *df* = 1405, *P* = 0.99, IR backgrounds). The blue opsin peak from Cos-1 cell extracts (416 nm; Chinen et al., 2003) was the same as the combined adult B-cone peaks from MSP, patch, and PIII model fit methods (one-sample *t*-test, *t* = −0.69, *df* = 8, *P* = 0.51, Table 2). The literature values for adult U-cone peaks averaged 361.0 ± 1.5 nm (s.e., *n* = 4). UV-cone signal levels were low in adult cone-PIII treatment-level datasets, and no peaks could be fit on adult roy or WT datasets on IR backgrounds. The spectral-model adult U-cone peaks on red backgrounds (348.8 ± 4.3 nm, s.e., *n* = 3220, Table 2) was not distinguishable from the mean of MSP or patch-recorded values (*t*-test, *t* = 0.10, *df* = 3222, *P* = 0.92). The 5–12 dpf larval U-cone peaks on IR backgrounds (roy: 373.1 ± 1.6 nm, s.e., *n* = 6640; WT, 360.0 ± 1.7 nm, s.e., *n* = 5148, Table 3) were the same as the literature values for adults (roy larvae: *t*-test, *t* = 0.19, *df* = 6642, *P* = 0.85; WT larvae: *t*-test, *t* = 0.02, *df* = 5147, *P* = 0.99). Combined peaks from adult MSP, patch, and adult and larval PIII model fit methods (360.9 ± 2.8 nm, *n* = 7, Tables 2 and 3) were at slightly longer wavelengths than Cos-1 cell extracts (355 nm) but not significant (one-sample *t*-test, *t* = 2.11, *df* = 7, *P* = 0.08). In summary, model-fit opsin peaks from cone-PIII spectral datasets are in excellent agreement with measurements previously made on individual cones in adult zebrafish, and many, but not all, of the opsin expression system values. The spectral model accurately extracts cone spectral peaks from PIII ERG spectral datasets that combine signals from multiple cone types.

**Table 3.** Present and literature values for larval zebrafish cone opsin absorbance peaks

Larval cone opsin peaks (nm)		Cone type			
Author	Method	UV	Blue	Green	Red
Nawrocki et al. (1985)	MSP	nd	414	478	538
Chinen et al. (2003)	Cos-1 express	355	416	467/476	548
Spectral model [means ± s.e. (*n*)]	Cone PIII *roy orbison*	373 ± 1.6 (6440)^a^	443 ± 1.7 (6440)^a^	459 ± 3.8 (6440)^a^	557 ± 0.9 (6440)^a^
	Cone PIII WT	360 ± 1.7 (5145)^a^	440 ± 2.3 (5145)^a^	460 ± 3.2 (5145)^a^	556 ± 1.2 (5145)^a^
			427 ± 6.7 (939)^b^	476 ± 6.8 (939)^b^	

*Note:* Microspectrophotometry (MSP), extracts of transfected Cos-1 cells (Cos-1 express), spectral model analysis of cone PIII peaks (Spectral model). Data from 5–12 dpf are combined as single, normalized, treatment-level datasets before fitting to the model. Cone PIII is the ERG signal amplitude remaining in aspartate perfused retinas. WT and *roy orbison* are zebrafish strains, the latter a mutant (D’Agati et al., 2017). (*n*), the number of data points fit by the Spectral model.

a IR background (*λ* > 780 nm).

b Red background (627 nm).

## Discussion

### Importance and limitations of the model

Model fits to treatment-level spectral datasets allow access to cone spectral peaks in intact functioning retinas. They measure *in situ* cone properties that might not be easily accessed by single-cell methods. Model fits are bulk measures, that is, only the opsin peaks expressed in significant fractions of the cone population can be measured. There are eight known zebrafish opsins (Chinen et al., 2003; Vihtelic et al., 1999), and all of these may be expressed, perhaps selectively, in different cone populations (Takechi & Kawamura, 2005; Tsujimura et al., 2007, 2010). Less commonly expressed opsins might be difficult to detect by the treatment-level spectral dataset method, while at the same time, potentially being important to color-processing neural circuits that would provide zebrafish with an unimaginably rich spectral world. We have combined modeling with chromatic adaptation to extend the range of detectable cone types that operate within a spectral waveband and to determine whether cone signals are composite peaks from mixed opsin cones or opsins expressed in separate cone populations.

One of the principal successes in the present study is identifying cone spectral peaks in larvae (Table 3), and the expected and unexpected ways in which they differ from adult cone peaks. Another is the confirmation of the predicted dual R1–R2 cone physiologies in the red waveband of adult zebrafish. The spectral modeling approach leads to a cross-check on molecular beliefs concerning opsin types in zebrafish and emphasizes the need to take a closer look at larval opsins, particularly the blue (SWS2) opsin. The method has a logical extension for studies of mutations in the development of retinal cone types, where it may be more efficient than single-cell techniques, particularly where population differences would require a very large number of measurements to detect subtle changes. Application of an ERG paradigm to measure cone properties could also have applications to current and future animal and human studies. This approach may prove valuable in profiling a species whose cone spectra are unknown, patients with uncharacterized opsin mutations, or patients suffering from retinal disease that shows selective cone loss.

### UV opsin peaks

Vision scientists came late to the idea that vertebrates had U-cones (Chen et al., 1984; Goldsmith, 1980; Hárosi & Hashimoto, 1983). Like other opsins, UV opsins are, across species, spectrally broad in peak distributions. UV opsins (SWS1-like sequences) are common to fish, birds, reptiles, amphibians, and mammals, including both rodents and humans (Shichida & Matsuyama, 2009). In mouse and rodents, UV opsin is maximally sensitive at 359 nm (Jacobs et al., 1991); however, the peak sensitivity has diverged toward longer wavelengths in other mammals, such as humans, 414 nm (Fasick et al., 1999); monkey, 430 nm (Baylor et al., 1987); cat, 440–450 nm (Daw & Pearlman, 1970; Guenther & Zrenner, 1993; Nelson, 1985; Nelson et al., 1985); and rabbit, 465 nm (Caldwell & Daw, 1978). The zebrafish U-cone peak at ∼360 nm is of a short-wavelength type and the peak remains constant throughout development, although the fraction of signal represented by U-cones changes, being large in larvae, but of diminished amplitude in adults. WT U-cone peaks *in situ* lie close to zebrafish SWS1 opsin values in solution (Chinen et al., 2003), while roy fit values may lie at somewhat longer wavelengths.

### Two blue opsins

There is only one gene for blue opsin in zebrafish, *SWS2*, which is continuously expressed in B-cones throughout development and into adulthood (Chinen et al., 2003; Takechi et al., 2008). A single blue-opsin antibody stains both the larval and adult long single B-cones (Vihtelic et al., 1999). A well-known B-cone reporter line expresses fluorescent protein in both larvae and adults (Takechi et al., 2008). It was not expected that larval and adult B-cone peaks would differ or that more than one B-cone peak would be found in WT larvae. One explanation might be a chromophore shift from vitamin A_2_ to vitamin A_1_ during development. When a shift to vitamin A_2_ was thyroxin-induced in adult zebrafish, MSP did not reveal any shift in B-cone spectral peaks. It was only rod, R-cone and G-cone peaks that shifted to longer wavelengths (Allison et al., 2004). The initial finding of a 416-nm peak for zebrafish SWS2 opsin extract was itself unexpected (Chinen et al., 2003). There were no comparable values in related species; SWS2 opsin peaks were commonly ∼440 nm (Chinen et al., 2005), as in species like goldfish, as measured by similar methods with a vitamin A_1_ reconstituted pigment (Chinen et al., 2005). Most amphibian SWS2 opsin peaks, as pigments reconstituted *in vitro* with vitamin A_1_, lie between 430 and 440 nm (Takahashi & Ebrey, 2003). In birds (zebra finch), the absorbance peak of SWS2 opsin is 440 nm (Yokoyama et al., 2000); among mammals, only the platypus expresses SWS2, with an absorbance peak at 451 nm (Davies et al., 2007). The current results suggest that there is *in vivo* a B2-cone peak in zebrafish larvae at ∼440 nm, like other species, but that the predominant peak shifts to the outlier value of 410–415 nm in adulthood. In WT larvae both B1 cone and B2 cone peaks are seen, extending the number of cone types expected in the blue waveband from one to two.

Within various opsin types, as grouped by sequence homology, migration of peak wavelengths is common (Jacobs, 2017) and is attributed in part to amino acid substitutions occurring on an evolutionary, not developmental, time frame (Chinen et al., 2005; Takahashi & Ebrey, 2003). We speculate that the different larval and adult B-cone peaks might involve isoforms of SWS2 opsin.

### Green opsins

While there is one adult G-cone morphology in zebrafish, the accessory members of double cones, or in larvae, long-single cones (Allison et al., 2010), current results suggest more than one G-cone spectral type in the green waveband. The four G-cone opsins are RH2 types, related to rhodopsin (RH1). In WT, peaks for at least three different G-cones appear in larval or adult physiology, suggesting a single cone morphology may choose one of the several spectral forms.

The developmental patterns of G-cone, RH2 mRNA expression, in zebrafish predict a physiological shift from shorter- to longer-wavelength G-cones during development (Takechi & Kawamura, 2005). In the current study, a G-cone peak shift was noted in the roy strain, from larval G1 cone peaks at 460 nm, similar to RH2-1 opsin, to adult G3 cone peaks at 482–488 nm, similar to RH2-2 or RH2-3 opsins. Selective chromatic adaptation did not change the roy adult or larval G-cone peaks but did change them in both WT larvae and WT adults, suggesting that multiple G-cones are active in WT at both adult and larval stages. In both roy and WT larval strains, the 460 nm G1 cone was the physiologically more sensitive type, and the type that disappeared in adulthood. The longer-wavelength, lower sensitivity larval WT G3 cones might be thought of as an early appearance of the adult G3 cone. Adult WT G-cones showed peak shift with chromatic adaptation, perhaps from a G3- to a G4-cone, the latter peaking at 495 nm with blue adaptation. Overall, WT shows a richer expression of G-cone types than roy. Expression of multiple RH2 opsins is well documented in WT larvae and adults (Tsujimura et al., 2007).

### Dual red opsins

In the long-wavelength, R-cone waveband, selective chromatic adaptation suggests not just one R-cone, but both R1 and R2 types. R1 and R2 cones form separate adaptation pools, so that the R-cone spectral peak can shift on red *vs.* blue backgrounds. A single R-cone expressing both L-opsins might have difficulty generating a spectral shift, as two L-opsins would share a single phototransduction pathway. Zebrafish and old-world primates might share color circuitry based on two separate cone types expressing highly homologous, long-wavelength opsins. In old-world primates, long-wavelength-sensitive (LWS) and middle-wavelength-sensitive (MWS) cones express opsins that are nearly identical by sequence homology, both belonging to the phylogenetic L-opsin class. In both zebrafish and old-world primates, these L-opsin genes are in a tandem array governed by a single promoter (Chinen et al., 2003; Jacobs & Neitz, 1987; Nathans et al., 1986). In both species, cones activate either one or the other opsin gene, but not both. In zebrafish, there is a spatial expression pattern. In adults, *LWS1*-activating R1 cones occur mainly in peripheral, ventral-nasal retina, whereas *LWS2*-activating R2 cones occur in central retina, although there is also spatial overlap among these types (Tsujimura et al., 2010). In old-world primates, the spatial distribution pattern of the corresponding MWS and LWS cones on the retina appears random (Roorda & Williams, 1999). The present results confirm physiological activity of R1 and R2 cone types in zebrafish, perhaps analogous to LWS and MWS primate cones.

In detergent extracts, LWS2 was found to be the shorter-wavelength-peaking red opsin and LWS1 was found to be the longer (Chinen et al., 2003). As LWS2 mRNA is heavily expressed in larvae (Takechi & Kawamura, 2005), current results suggest an *in situ* R2-cone, LWS2 opsin peak at 551–554 nm, as seen in two R2-cone peaks for two larval strains on red chromatic backgrounds. In adults, the mean of non-red-adapted R1 cone peaks is 572 nm. This estimate is perhaps a lower bound of the *in situ* LWS1 opsin peak, as this R1-cone signal may contain an admixture of the shorter-wavelength R2 cone signal.

The discrepancy between the 558 nm LWS1 opsin peaks as measured in solution extract (Chinen et al., 2003) and the longer-wavelength adult R-cone peaks found by MSP was previously noted (Endeman et al., 2013), including a suggestion that possibly a combination of longer-wavelength-absorbing vitamin A_2_ with shorter-wavelength vitamin A_1_ red-opsin pigments, all in the same cone cell, was a potential explanation (Endeman et al., 2013). Later, the cytochrome cyp27c1 was found to mediate the production of vitamin A_2_, but MSP of R-cones in cyp27c1 mutant fish showed the same spectral peaks as R-cones in WT fish, suggesting that in zebrafish, vitamin A_2_ is not normally made and does not contribute to the positioning of R-cone peaks (Enright et al., 2015). Further, the mixing of vitamin A_1_ and vitamin A_2_ in individual R-cones might not lead to separate adaptation pools for the two opsins, as found here. We speculate that the long-wavelength shifts for *in situ*, as compared with *in solution*, LWS1 opsins is probably methodological.

### Strain variations in cone types

There were differences in cone spectral signals between *roy orbison* and WT zebrafish strains. U-cone signals were lower amplitude in roy larvae than in WT and exhibited a longer wavelength peak. In roy larvae, there were five physiological cone types, but in WT larvae, there were seven. In roy adults, there were four physiological spectral peaks, but in WT adults, there were six, with roy U-cone signals entirely absent. The recessive *roy orbison* spontaneous mutation was recently identified in *MPV17*, a gene coding a mitochondrial membrane protein (D’Agati et al., 2017). The phenotypes of *MPV17* mutations are species dependent; in mouse and humans, they cause genetic disorders of kidney and liver, respectively, but in zebrafish, a loss of iridophores, producing an otherwise healthy, roy coloration variant (D’Agati et al., 2017). Zebrafish cones are dense with mitochondria in the ellipsoid region. These support the high cone metabolic activity (Giarmarco et al., 2017; Tarboush et al., 2012). In retrospect, a mitochondrial mutation might well affect cones and perturb patterns of cone development. The roy coloration mutation is likely altered in visual system features.

### Roles of authors

RFN, assembled equipment, wrote code, experimental design, managing author; AB, larval datasets, dataset analysis; TS, adult datasets, dataset analysis; MT, adult datasets, dataset analysis; SSP, experimental design, larval datasets, spectral modeling, code writing.
